# Prognostic performance of preoperative cardiac troponin and perioperative changes in cardiac troponin for the prediction of major adverse cardiac events and mortality in noncardiac surgery: A systematic review and meta-analysis

**DOI:** 10.1371/journal.pone.0215094

**Published:** 2019-04-22

**Authors:** Caroline A. S. Humble, Stephen Huang, Ib Jammer, Jonas Björk, Michelle S. Chew

**Affiliations:** 1 Department of Anesthesiology and Intensive Care, Medical and Health Sciences, Linköping University, Linköping, Sweden; 2 Department of Intensive Care Medicine, The University of Sydney, Nepean Hospital, Sydney, Australia; 3 Department of Clinical Medicine, University of Bergen, Bergen, Norway; 4 Department of Anaesthesia and Intensive Care, Haukeland University Hospital, Bergen, Norway; 5 Division of Occupational and Environmental Medicine, Lund University, Lund, Sweden; 6 Clinical Studies Sweden, Forum South, Skåne University Hospital, Lund, Sweden; Keele University, UNITED KINGDOM

## Abstract

**Background:**

Increased postoperative cardiac troponin (cTn) independently predicts short-term mortality. Previous studies suggest that preoperative cTn also predicts major adverse cardiovascular events (MACE) and mortality after noncardiac surgery. The value of preoperative and perioperative changes in cTn as a prognostic tool for adverse outcomes has been sparsely investigated.

**Methods and findings:**

A systematic review and meta-analysis of the prognostic value of cTns for adverse outcome was conducted. Adverse outcome was defined as short-term (in-hospital or <30 days) and long-term (>30 days) MACE and/or all-cause mortality, in adult patients undergoing noncardiac surgery. The study protocol (CRD42018094773) was registered with an international prospective register of systematic reviews (PROSPERO). Preoperative cTn was a predictor of short- (OR 4.3, 95% CI 2.9–6.5, p<0.001, adjusted OR 5.87, 95% CI 3.24–10.65, p<0.001) and long-term adverse outcome (OR 4.2, 95% CI 1.0–17.3, p = 0.05, adjusted HR 2.0, 95% CI 1.4–3.0, p<0.001). Perioperative change in cTn was a predictor of short-term adverse outcome (OR 10.1, 95% CI 3.2–32.3, p<0.001). It was not possible to conduct pooled analyses for adjusted estimates of perioperative change in cTn as predictor of short- (a single study identified) and long-term (no studies identified) adverse outcome. Further, it was not possible to conduct pooled analyses for unadjusted estimates of perioperative change in cTn as predictor of long-term adverse outcome, since only one study was identified. Bivariate analysis of sensitivities and specificities were performed, and overall prognostic performance was summarized using summary receiver operating characteristic (SROC) curves. The pooled sensitivity and specificity for preoperative cTn and short-term adverse outcome was 0.43 and 0.86 respectively (area under the SROC curve of 0.68). There were insufficient studies to construct SROCs for perioperative changes in cTn and for long-term adverse outcome.

**Conclusion:**

Our study indicates that although preoperative cTn and perioperative change in cTn might be valuable predictors of MACE and/or all-cause mortality in adult noncardiac surgical patients, its overall prognostic performance remains uncertain. Future large, representative, high-quality studies are needed to establish the potential role of cTns in perioperative cardiac risk stratification.

## Introduction

### Background

Cardiac morbidity and mortality are common complications [1, 2] to the large number of noncardiac surgeries carried out on adult patients every year [3, 4]. Current guidelines recommend the use of the RCRI [2] and NSQIP-MICA [5] for cardiovascular risk stratification in noncardiac surgery [6–9]. The discriminative ability and generalizability of these indices have been questioned [5, 6, 10] and there is a need for new and, better tools to stratify patients according to risk of perioperative cardiac morbidity and mortality. Cardiac troponin I and T (cTnI/T) are cardiac specific proteins released by cardiomyocytes into the blood following injury to the myocardium that may or may not be due to ischemia (e.g. severe sepsis) [11]. The prognostic value of postoperative cTn has previously been investigated by large, multicenter prospective studies [1, 12] and in two systematic reviews and meta-analyses [13, 14]. Guidelines now suggest postoperative surveillance for myocardial injury in high-risk patients [6, 7, 9]. In contrast, preoperative cTn and perioperative changes in cTn (i.e. change between pre- and postoperative cTn) are still sparsely evaluated. The association between preoperative troponin levels and short-term major adverse cardiovascular events (MACE) and mortality was demonstrated in a recent meta-analysis [15]. However, data on long-term adverse outcome was limited in that study. Changes in perioperative troponins are also relevant to investigate since they may provide an early indication of perioperative myocardial injury that is not reflected in preoperative or postoperative values alone. As far as we are aware, the predictive value of perioperative change in cTn has not been analyzed in a systematic review.

Although meta-analyses may summarize the prognostic value of biomarkers, the relationship between sensitivity and specificity is not considered and there is a need to assess the overall prognostic performance of the pooled results. This may be best done by considering cTns as a test of diagnostic accuracy.

### Objectives

The objective of our study was two-fold:

Firstly, to systematically review and conduct meta-analyses to answer the following questions: 1) Does preoperative cTn predict short- and long-term adverse outcome? 2) Do perioperative changes in cTn predict short- and long-term adverse outcome?

Secondly, we sought to analyze the overall prognostic performance of preoperative cTn and perioperative changes in cTn. The adverse outcome was short- (in-hospital or <30 days) and long-term (>30 days) all-cause mortality and/or MACE in adult patients undergoing noncardiac surgery.

## Methods

We adhered to the PRISMA (Preferred Reporting Items for Systematic Reviews and Meta-Analyses) statement [16] in conducting and reporting this systematic review. On the 23rd of January 2016, we conducted two searches in the electronic databases, Medline via PubMed and Embase via Ovid. No filters, with respect to year of publication or language, were used. The following MeSH terms were used in the Medline search: ‘Troponin’, ‘Surgical Procedures, Operative’/’surgery’/’Postoperative Complications’, ‘Cardiovascular Diseases’/’Mortality’/’Death’, ‘Prognosis’/’Risk Assessment’/’Sensitivity and Specificity’, ‘Perioperative Care’/’Perioperative Period’ (S1 File). The search strategy was developed with the assistance of a librarian at Lund University, Sweden. On the 23^rd^ of June 2017, we conducted an updated search identical to the two searches mentioned, to identify papers published between 23^rd^ of January 2016 to 23^rd^ of June 2017. Two authors independently screened titles (MSC, CH or MSC, IJ), abstracts and full-text articles (IJ, CH or IJ, MSC) in accordance with predefined eligibility criteria. Differing opinions on whether to include or exclude full-text articles were resolved through discussion by two authors (IJ, CH or IJ, MSC). If consensus could not be reached, a third author (MSC or CH) reviewed the full-text article and made the final decision. Furthermore, the reference lists of the included studies [12, 17–35], in addition to three central reviews [7, 36, 37] were screened to identify additional eligible studies. The study protocol (CRD42018094773) was registered with an international prospective register of systematic reviews (PROSPERO). (S2 File).

### Eligibility criteria

Full-text articles were included in our qualitative analysis if they fulfilled the following:

#### Inclusion criteria

Population: Human adults, i.e. ≥18 years old.
Type of surgery: Non-cardiac surgery.
Type of study: All studies measuring cTn, pre- and/or pre- and postoperatively, investigating the association between preoperative cTn and outcome(s) and/or perioperative change in cTn (i.e. change between pre- and postoperative cTn) and outcome(s).
Type of cardiac troponin: cTnT, cTnI, high-sensitivity cTnT (hs-cTnT), high-sensitivity cTnI (hs-cTnI).
Outcome: All-cause mortality and/or MACE as defined by the original studies.
Effect measure: Unadjusted odds ratio (OR); adjusted OR (aOR); unadjusted risk ratio (RR); adjusted RR (aRR); unadjusted hazard ratio (HR); adjusted HR (aHR); data available to construct 2x2 contingency table; single p-values (i.e. if nothing else is given); relevant quotes on association (i.e. if not stated in numbers, e.g. “the unadjusted association was not statistically significant”).
Blood sampling: Up to 30 days prior to and after surgery.

#### Exclusion criteria

Transplantation surgery.
Non-full text articles, not full report, case series, letters, brief reports.

### Bias assessment

We used the Quality In Prognostic Studies (QUIPS) tool [38] to assess the risk of bias in the individual, included studies. The QUIPS tool consists of six, separate bias domains: selection bias; attrition bias; prognostic factor (i.e. cTn) measurement bias; outcome measurement bias (i.e. eligible outcome); study confounding and finally, bias related to statistical analysis and reporting. We further customized the tool to suit our study (S3 File). Importantly, for the study confounder assessment we predefined six important, potential confounders: age [1]; RCRI score [2] (in any way it was adjusted for); pre-existing kidney disease [39] or injury; peripheral vascular disease [1]; urgency of surgery [1]; and length of surgery [40]. For each of the six domains the risk of bias was assessed as low, moderate or high. The bias assessment was conducted independently by two authors (CH, MSC). Differing opinions were resolved through discussion by the two authors.

### Data extraction

The following data extraction was performed from the included full-text articles independently by two authors into piloted forms:

Baseline data: First author, year of publication, study design, number of participating centers, study period, sample size (i.e. number of patients included in the statistical analysis), type of surgery, risk of surgery [9, 41], urgency of surgery, mean or median age (if not explicitly stated, we calculated it if possible), male proportion (if not explicitly stated, we calculated it if possible).
Troponin data: cTn type, assay manufacturer, timing and frequency of cTn sampling (i.e. for the cTn included as prognostic factor in the statistical analysis), prognostic cTn cut-off concentration (for conventional cTn we converted all units to μg/L).
Outcome data: Length of follow-up for eligible outcome (i.e. adverse outcome), proportion between number of patients lost to follow-up and number of patients at study baseline, eligible outcome, proportion of sample size with adverse outcome, proportion of patients with elevated cTn with adverse outcome, proportion of patients with non-elevated cTn with adverse outcome, data to reconstruct 2x2 contingency table (S1 Table), sensitivity and specificity (if not explicitly stated, we calculated it if possible), eligible effect measure with 95% CI and p-value (if reported), variables adjusted for in multivariate analysis (if applicable).

We extracted all eligible data reported, e.g. if a study reported more than one eligible effect size, we extracted all eligible effect sizes.

### Statistical analysis

Meta-analyses were subgrouped according to: type of predictor (i.e. preoperative cTn or perioperative change in cTn), type of effect measure (e.g. OR or HR), whether the effect size was unadjusted or adjusted and lastly timing of adverse outcome. Furthermore, if possible we conducted subgroup meta-analysis according to the type of troponin assay (i.e. cTnT/cTnI or hs-TnT). Regarding timing of adverse outcome, we adopted a limit of 30 days or less, including in-hospital adverse outcome, for short-term adverse outcome and more than 30 days for long-term adverse outcome. If one study had more than one effect size eligible for the same sub group meta-analysis, the effect size with the most outcome events was chosen. If there was no difference in number of outcome events we chose the effect size reported in the abstract of the study. Unadjusted OR reported in the meta-analyses were calculated from 2x2 contingency tables when possible, even for studies that reported a corresponding unadjusted OR. If the study, on the other hand, reported an unadjusted OR, but not data enough to reconstruct a 2x2 contingency table, the OR reported in the study was used in the pooled analysis. We pooled effect sizes using the DerSimonian and Laird random effects model [42]. All p-values were two-sided and a value <0.05 was considered statistically significant. In seven of the eight meta-analyses the generic inverse variance method was used, which required the standard error of the natural logarithm of the effect size calculated according to Woolf’s formula [43]. We calculated the I^2^ statistic and Cochran’s Q (reported with a p-value) to assess heterogeneity between studies. An I^2^ value more than 25 per cent and a p-value less than 0.10 was considered to represent significant heterogeneity [44]. Analyses were performed using MedCalc Statistical Software version 18.5.0 and 18.11.3 (MedCalc Software bvba, Ostend, Belgium, https://www.medcalc.org; 2016). Sensitivity and specificities for each study were summarized in forest plots, demonstrating between-study variation. The sensitivities and specificities were pooled and the mean was estimates by bivariate modelling [45]. In order to evaluate the overall prognostic performance of cTn, we generated summary receiver operating characteristic (SROC) curves (R software, v3.5.0).

## Results

**Fig 1:**
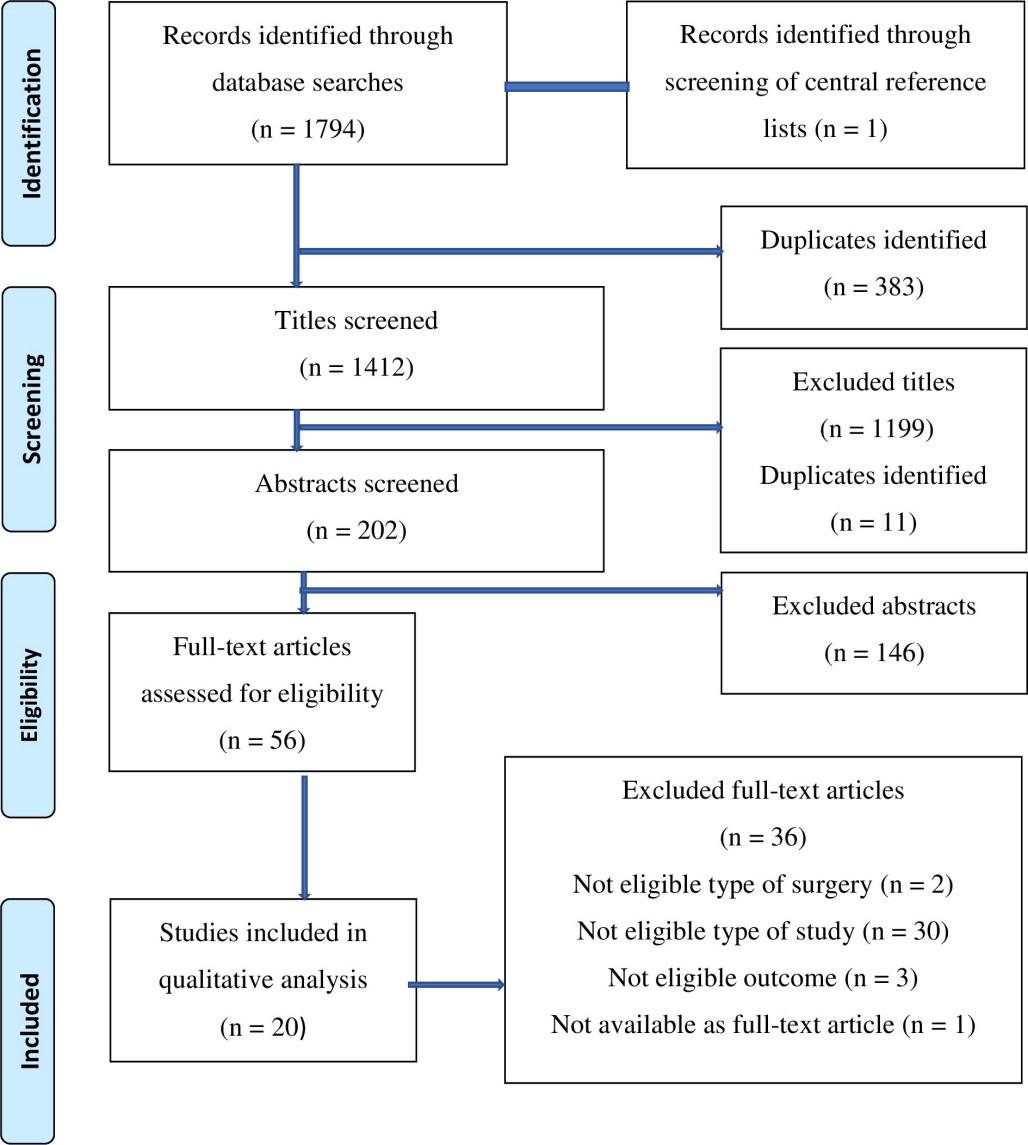
Flow diagram of study identification and selection.

Our search strategy identified a total of 1795 records. After an initial screening of titles and abstracts, 1739 records were eliminated, of which 394 were duplicates. Fifty-six full-text articles were assessed for eligibility. Twenty eligible studies [12, 17–35] were identified. (Fig 1). Nineteen [17–35] studies were included addressing preoperative cTn and three studies [12, 26, 29] addressing perioperative change in cTn as prognostic factor. Notably Gillman et al. [29] and Nagele et al. [26] were included in both categories.

Twelve studies provided adjusted estimates [12, 19, 23–29, 31, 34, 35]. In the remaining studies only unadjusted estimates were provided. We wrote to individual authors requesting additional data in order to obtain original data to conduct our own adjusted analyses but received inadequate responses, consequently this option was not further explored. Results are therefore presented for adjusted and unadjusted analyses for preoperative and perioperative changes in cTns respectively.

### Bias assessment

Table 1 provides an overall picture of the methodological quality of the studies as evaluated by the QUIPS tool [38]. A majority of the studies had moderate or high risk of selection bias [17, 19–21, 23, 25–28, 32, 34, 35], confounding [12, 17–22, 24–29, 31–35] and bias related to statistical analysis and presentation of results [17–23, 26, 27, 29, 32–35]. Furthermore, most of the studies evaluating adverse outcome other than ‘mortality’ had moderate risk of outcome measurement bias [17–19, 23, 25, 27, 29, 31, 33].

**Table 1:** Bias assessment with Quips tool [38].

Bias Domains						
First author, Year	Selection bias (likelihood that relationship between cTn and outcome is different for participants and eligible nonparticipants) (H/M/L)	Attrition bias (likelihood that relationship between cTn and outcome is different for completing and non-completing participants) (H/M/L)	cTn measurement bias (likelihood of differential measurement of of cTn related to the level of outcome) (H/M/L)	Outcome measurement bias (likelihood of differential measurement of outcome related to the baseline level of cTn) (H/M/L)	Confounding^t^ (likelihood that the effect of cTn is distorted by another factor that is related to cTn and outcome) (H/M/L)	Bias related to statistical analysis and presentation of results (H/M/L)
Münzer, 1996 [17]	H	L	L	M	H	M
Gibson, 2006 [18]	L	L	M	M	H	M
Oscarsson, 2009 [19]	M	H	L	M	H	H
Chong, 2010 [20]	M	L	L	L	H	M
Talsnes, 2011[21]	M	L	L	L	H	H
Alcock, 2012 [22]	L	L	L	L	H	M
Biccard, 2012 [23]	H	H	M	M	L	H
Degos, 2012 [24]	L	L	L	L	H	L
Chong, 2013 [25]	M	M	M	M	H	L
Nagele, 2013 [26]	H	L	L	L	M	M
Weber, 2013 [27]	H	L	L	M	M	M
Zheng, 2013 [28]	M	L	M	L	M	L
Gillmann, 2014 [29]	L	L	L	M	M	M
Hietala, 2014 [30]	L	L	L	L	L	L
Ma, 2015 [31]	L	L	L	M	M	L
Maile, 2016 [32]	H	L	M	L	M	M
Thomas, 2016 [33]	L	L	L	M	H	M
Zimmerman, 2016 [34]	H	L	L	L	H	M
Devereaux, 2017 [12]	L	L	L	L	M	L
Kopec, 2017 [35]	H	L	L	L	M	M

cTn = Cardiac troponin. H = High risk of bias. M = Moderate risk of bias. L = Low risk of bias. t = We defined the following factors as important, potential confounders: age; Revised Cardiac Risk Index Score; pre-existing kidney disease or injury; peripheral vascular disease; urgency of surgery; length of surgery.

### Preoperative cardiac troponin

#### Study and patient related characteristics

Nineteen studies assessed preoperative cTn with a total sample size of 13386 (range 33 to 4575) [17–35]. A majority of the studies were prospective cohort studies performed at a single centre [17–26, 28–31, 33, 35]. The studies included patients undergoing a wide range of non-cardiac surgeries, the majority of which were intermediate- or high- risk procedures. Six of the studies specifically included only patients with cardiovascular disease or patients at risk of it [17, 22, 26–28, 35]. One study [19] included only patients with ASA class III to IV. (Table 2).

**Table 2:** Study and patient related characteristics of included studies.

Studies assessing the association between **preoperative** cardiac troponin and adverse outcome							
First author, Year	Study design	Study period	Sample size^§^	Type, risk (low/intermediate/high) [41] and urgency of surgery	Mean age±SD	Male proportion in percentage	
Münzer, 1996 [17]	Prospective cohort. Single center.	April 1, 1992-March 31, 1993	139	Type: Non-cardiac surgery Risk: NR Urgency: Elective	70	75^†^	
Gibson, 2006 [18]	Prospective cohort. Single center.	April 2004-April 2005	44	Type: Major lower extremity amputation Risk: High^††^ Urgency: Elective	71^•^	64^†^	
Oscarsson, 2009 [19]	Prospective cohort. Single center.	April 15, 2007-April 14, 2008	186	Type: Non-cardiac surgery (urological, gynecological, orthopedic, ophthalmological, neurosurgical, reconstructive procedures) Risk: Low, intermediate^††^ Urgency: Emergent, urgent	NR for sample size	36^†^	
Chong, 2010 [20]	Prospective cohort. Single center. Sub study of RCT.	April 2008-February 2009	33	Type: Orthopedic surgery Risk: Intermediate^††^ Urgency: Emergent	85.8±9.6	33	
Talsnes, 2011 [21]	Prospective cohort. Single center.	2005–2009	146	Type: Hip fracture surgery Risk: Intermediate^††^ Urgency: NR	NR for sample size	NR for sample size	
Alcock, 2012 [22]	Prospective cohort. Single center.	January 2011-November 2011	352	Type: Major non-cardiac surgery (major vascular, major orthopedic, general, major urological, major neurosurgery, lower risk) Risk: High, intermediate, low^††^ Urgency: Elective	72.2±9.6	64	
Biccard, 2012 [23]	Prospective cohort. Single center.	February 2008-March 2011	534^***^	Type: Vascular surgery Risk: Intermediate, high^††^ Urgency: Elective	NR for sample size	NR for sample size	
Degos, 2012 [24]	Prospective cohort. Single center.	2003–2007	368	Type: Subarachnoid hemorrhage coiling Risk: Intermediate^††^ Urgency: NR	50±13	36	
Chong, 2013 [25]	Prospective cohort. Single center. Sub study of RCT.	April 2008-February 2009	187	Type: Orthopedic surgery Risk: Intermediate^††^ Urgency: Emergent	76.7±9.3	29^†^	
Nagele, 2013 [26]	Prospective cohort. Single center. Sub study of RCT.	March 2008-December 2011	608	Type: Vascular, orthopedic, ear-nose-throat, gynecology, urology, neurosurgery Risk: Intermediate, high^††^ Urgency: Elective	64.8^†^	62^†^	
Weber, 2013 [27]	Prospective cohort. Multicenter.	2006–2009	979	Type: Major non-cardiac surgery (abdominal, urological, orthopedic, gynecologic, neck, vascular) Risk: Intermediate, high^††^ Urgency: Non-emergent	69±8	54	
Zheng, 2013 [28]	Prospective cohort. Single center.	January 2010-March 2012	380	Type: Non-cardiac surgery Risk: Intermediate, high Urgency: Elective	65.3	46^†^	
Gillmann, 2014 [29]	Prospective cohort. Single center.	4-year period until October 2012	455	Type: Open aortic, peripheral vascular, or carotid surgery Risk: High^††^ Urgency: Elective	NR	NR	
Hietala, 2014 [30]	Prospective cohort. Single center.	October 19, 2009-May 19, 2010	200	Type: Low-trauma hip fracture surgery Risk: Intermediate^††^ Urgency: NR	80.8^•^	34	
Ma, 2015 [31]	Prospective cohort. Single center.	December 2007-December 2013	2519	Type: Non-cardiac surgery (abdominal, gynecological, urological, orthopedic, reconstructive, vascular) Risk: Intermediate, high^††^ Urgency: Emergent	77.3±8.4	52	
Maile, 2016 [32]	Retrospective cohort. Single center.	March 1, 2006-June 5, 2013	4575	Type: Non-cardiac surgery (general, neurosurgery, obstetrics/gynecology, oral/maxillofacial, orthopedics, otolaryngology, plastics, thoracic, transplantation, urology, vascular) Risk: Low, intermediate, high^††^ Urgency: Non-emergent	63^•^	55	
Thomas, 2016 [33]	Prospective cohort. Single center. Sub study of RCT.	NR	85	Type: Major vascular procedure (open intra-abdominal, open extra-abdominal lower limb reperfusion, endovascular AAA repair) Risk: High^††^ Urgency: Elective	74±8	72	
Zimmerman, 2016 [34]	Retrospective review. Two centers.	January 2008-December 2014	464	Type: General surgery Risk: NR Urgency: Emergent	69.8^†^	51^†^	
Kopec, 2017 [35]	Prospective cohort. Single center. Sub study of RCT.	March 2008-December 2011	572	Type: Major non-cardiac surgery (vascular, orthopedic, ear-nose-throat, gynecology, urology, neurosurgery Risk: Intermediate, high^††^ Urgency: Elective	64.9±10.7	62	
Studies assessing the association between **perioperative change** in cardiac troponin and adverse outcome							
First author, Year	Study design	Study period	Sample size	Type, risk (low/intermediate/high) [41] and urgency of surgery	Mean age±SD	Male proportion in percentage	
Nagele, 2013 [26]	Prospective cohort. Single center. Sub study of RCT.	March 2008-December 2011	608	Type: Vascular, orthopedic, ear-nose-throat, gynecology, urology, neurosurgery Risk: Intermediate, high^††^ Urgency: Elective	64.8^†^	62.5^†^	
Gillmann, 2014 [29]	Prospective cohort. Single center.	4-year period until October 2012	455	Type: Open aortic, peripheral vascular, or carotid surgery Risk: High^††^ Urgency: Elective	NR	NR	
Devereaux, 2017 [12]	Prospective cohort. Multicenter.	October 2008-December 2013	7857	Type: Major vascular, major general, major thoracic, major urology, major gynecology, major orthopedic, major neurosurgery, low risk surgery Risk: Low, intermediate, high^††^ Urgency: Elective, urgent, emergent	NR for sample size	NR for sample size

AAA = Abdominal aortic aneurysm. NR = Not reported. RCT = Randomized controlled trial. SD = Standard deviation.

§ = Patients included in eligible effect measure analysis.

† = Not explicitly stated, calculated by authors.

†† = Not explicitly stated, concluded by authors.

• = Median age.

***Discrepancy between reported figures at different locations in the article.

#### Cardiac troponin related characteristics

Conventional cTnI or cTnT was used in most of the studies, whereas hs-cTnT was used in the remaining studies [22, 26, 27, 29, 33, 35]. The range of prognostic cut-off concentration for conventional cTnT and cTnI was 0.03 μg/L to 0.2 μg/L and 0.05 μg/L to 0.5 μg/L, respectively. For hs-cTnT the range was 14 ng/L to 17.8 ng/L. Two studies did not quantify their cut-off concentrations [18, 28]. The timing of preoperative blood sampling ranged from a few days to hours before surgery, and was not specified in six studies [17, 20, 25, 29, 33, 34]. Assay characteristics, cut-off concentrations and timing of cTn sampling are summarized in S2 Table.

#### Ability of preoperative cardiac troponin to predict short-term adverse outcome—unadjusted analysis

**Fig 2:**
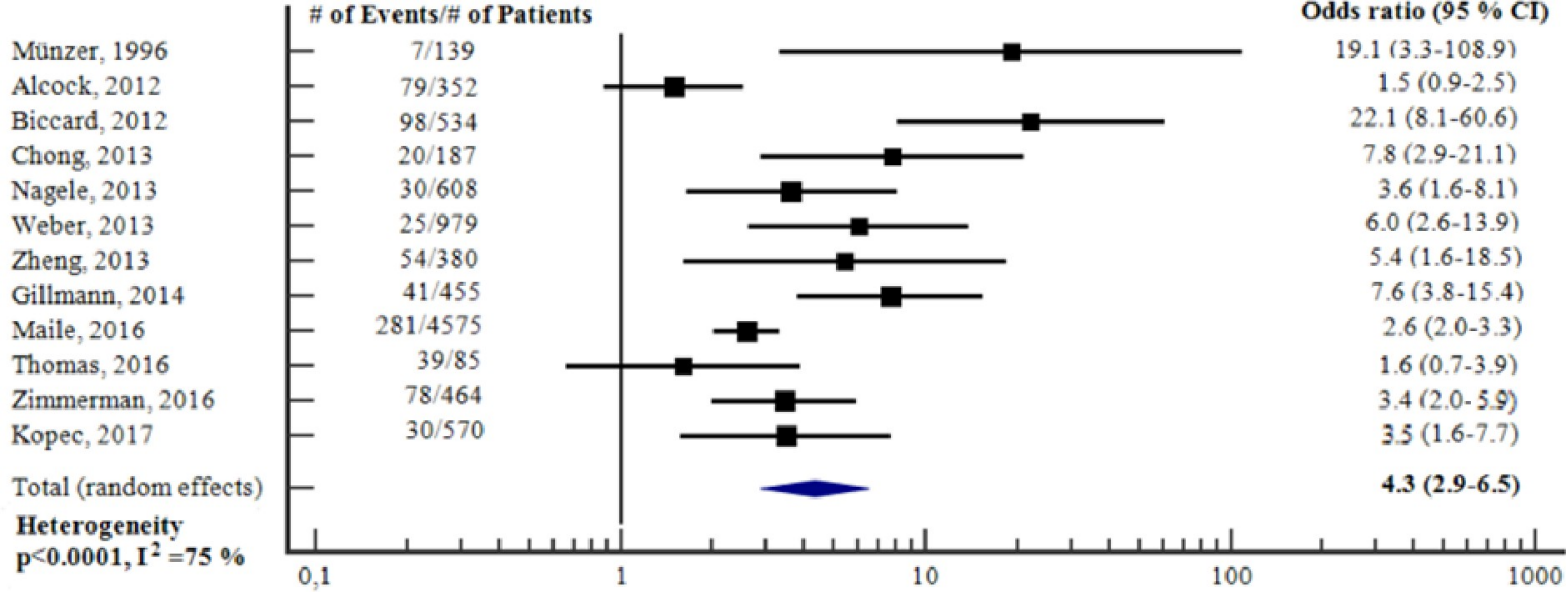
Unadjusted odds ratios for elevation in preoperative cTn to predict short-term adverse outcome. Forest plot showing the individual and pooled unadjusted odds ratios from the included studies. cTn = Cardiac troponin. CI = Confidence interval. # = Number of.

The proportion of patients who had short-term adverse outcome varied from 3 to 46 per cent. The sensitivity and specificity for preoperative cTn to predict short-term adverse outcome ranged from 9 to 70 and 60 to 99 per cent, respectively. (Table 3). Fig 2 reports the meta-analysis of the twelve studies [17, 22, 23, 25–29, 32–35] for which we could obtain an OR for preoperative cTn to predict short-term adverse outcome. The total sample size was 9328 (range 85 to 4575). Preoperative cTn was a significant, unadjusted predictor of short-term adverse outcome (OR 4.3, 95% CI 2.9–6.5, p<0.001), however there was substantial heterogeneity between the studies (I^2^ = 75%, p<0.0001). Subgroup meta-analyses, according to the type of troponin assay (i.e. conventional cTnT/cTnI or hs-cTnT), showed that cTn was clearly predictive in both subgroups of studies (S1 and S2 Figs).

**Table 3:** Association between cardiac troponin and adverse outcome.

Association between **preoperative** cardiac troponin and adverse outcome										
First author, Year	Length of follow-up	No. lost to follow-up/No. patients^§§^	Adverse outcome	No. events/Sample size^§^ (%)	No. events/No. elevated cTn	No. events/No. non-elevated cTn	Sensitivity, Specificity	Unadjusted OR/HR; 95% CI; p-value	Adjusted OR/HR; 95% CI; p-value	Variables adjusted for in multivariate analysis
Münzer, 1996 [17]	1. 3 days 2. 3 days	0^††^/139	1. Re-MI 2. Left ventricular failure	1. 6/139 (4) 2. 7/139 (5)	1. 2/8 2. 3/8	1. 4/131 2. 4/131	1. 33, 95 2. 43, 96	1. NR; NR; <0.05°/OR 10.6; 1.6–69.7; 0.01°° 2. NR; NR; <0.05°/OR 19.1; 3.3–108.9; 0.0009°°	1. NR; NR; NR 2. NR; NR; NR	1. NA 2. NA
Gibson, 2006 [18]	6 weeks	0^††^/44	Cardiac events (non-fatal MI, cardiac death)	10/44 (23)	3/3	7/41	30, 100	NR; NR; 0.009°/OR 32.2; 1.5–691.3; 0.03°°	NR; NR; NR	NA
Oscarsson, 2009 [19]	1. 30 days 2. 30 days 3. 3 months	25/211	1. MACE (AMI and/or cardiovascular death) 2. Mortality 3. Mortality	1. 26/186 (14) 2. 23/186 (12) 3. 43/186 (23)	1. NR/40 2. NR/40 3. NR/40	1. NR/146 2. NR/146 3. NR/146	1. NR, NR 2. NR, NR 3. NR, NR	1. NR; NR; NR 2. NR; NR; >0.10 3. NR; NR; >0.10	1. OR 4.8; 1.5–15.5; 0.008 2. NR; NR; NR 3. NR; NR; NR	1. Age, IHD, CHF, creatinine clearance, RCRI, malignancy, diuretics, organic nitrates, preoperative NT-proBNP >1800 pg/ml. 2. NA 3. NA
Chong, 2010 [20]	6 months	0^††^/33	Mortality	13/33 (39)	3/11	10/22	23, 60	OR 0.41; 0.09–2.00; 0.272°/OR 0.45; 0.1–2.2; 0.3°°	NR; NR; NR	NA
Talsnes, 2011 [21]	3 months	0^††^/146	Mortality	NR for sample size	NR/NR	NR/NR	NR, NR	OR 10.9; 2.2–54.0; 0.003	NR; NR; >0.05	Age, sex, ASA physical status, CK-MB/CK-ratio
Alcock, 2012 [22]	In-hospital	0^††^/352	Myocardial necrosis (hs-cTnT ≥14 ng/L and ^Δ^hs-cTnT≥50%)	79/352 (22)	NR/109	NR/243	NR, NR	OR 1.50; 0.89–2.54; 0.127	NR; NR; NR	NA
Biccard, 2012 [23]	30 days	26/560	MACE (death, cTnT or cTnI>URL within the first 3 postoperative days)	98/534 (18)	20/25	78/509	20, 99	OR 22.1; 8.1–60.0; <0.001°/OR 22.1; 8.1–60.6; <0.0001°°	OR 57; 6–496; <0.001	RCRI, preoperative BNP, preoperative CRP
Degos, 2012 [24]	1 year after ICU discharge	0^††^/368	Mortality	64/368 (17)	31/80	33/288	48, 84	NR; NR; NR°/OR 4.9; 2.7–8.7; <0.0001°°	OR 2.29; 1.08–4.86; 0.03	Seizure, Fisher score, intraventricular hemorrhage, hydrocephalus, male, age, GCS, S100β >5 µg/L
Chong, 2013 [25]	In-hospital	0^††^/187	Cardiac events (AMI, CHF, new onset or rapid AF, major arrhythmia, cardiac arrest)	20/187 (11)	NR/29	NR/158	NR, NR	OR 7.8; 2.9–21.1; <0.001	OR 7.4; 2.3–24.2; <0.001	Preoperative ECG changes
Nagele, 2013 [26]	1. 72 h 2. 3 years	17/625	1. AMI 2. Mortality	1. 30/608 (5) 2. 80/608 (13)	1. 21/247 2. NR/247	1. 9/361 2. NR/361	1. 70, 61 2a. NR, NR 2b. NR, NR	1. OR 3.67; 1.65–8.15; 0.001°/OR 3.6; 1.6–8.1; 0.002°° 2a. NR; NR; NR 2b. NR; NR; NR	1. NR; NR; NR 2a. HR 2.11; 1.26–3.53; 0.004 2b. HR 2.17; 1.19–3.96; 0.011	1. NA 2a. Age, sex 2b. Age, sex, race, eGFR, history of CAD, hypertension, diabetes
Weber, 2013 [27]	1. In-hospital 2. In-hospital	0^††^/979	1. Mortality 2. Combined endpoint (mortality, AMI, cardiac arrest, VF, CPR, acute decompensated heart failure)	1. 25/979 (3) 2. 36/979 (4)	1. 16/233 2. NR/233	1. 9/746 2. NR/746	1. 64, 77 2. NR, NR	1. NR; NR; NR°/OR 6.0; 2.6–13.9; <0.0001°° 2. HR 3.73; 1.90–7.31; 0.0001	1. NR; NR; NR 2. HR 2.60; 1.27–5.31; 0.0088	1. NA 2. RCRI ≥2, NYHA class II-IV, systolic blood pressure
Zheng, 2013 [28]	In-hospital^††^	0^††^/380	Adverse cardiac events (acute myocardial ischemia, AMI, malignant arrhythmia, CHF, cardiac death)	54/380 (14)	5/11	49/369	9, 98	OR 5.44; 1.60–18.51; 0.007°/OR 5.4; 1.6–18.5; 0.007°°	OR 8.78; 1.43–53.71; 0.019	Age, race, abnormal ECG at baseline, myocardial infarction history, baseline HO-1
Gillmann, 2014 [29]	30 days	0^††^/455	MACE (MI type I/II, cardiovascular death, any new rise in cTn prompted by clinical suspicion for ACS with cut-offs cTnT>0.05 µg/L and hs-cTnT>50 ng/L)	41/455 (9)	28/119	13/336	68,78	NR, NR, NR°/OR 7.6; 3.8–15.4; <0.0001°°	‘independently associated’	NR
Hietala, 2014 [30]	1. 30 days 2. 1000 days	1. 4/200 2. 4/200	1. Mortality 2. Mortality	1. 18/200 (9) 2. NR/200	1. NR/36 2. NR/36	1. NR/160 2. NR/160	1. NR, NR 2. NR, NR	1. NR; NR; NR 2. NR; NR; NR	1.‘independent predictor’ 2. HR 1.95; 1.20–3.15; 0.007	1. Age, renal impairment, dementia or AF, red blood cell transfusions, new ECG changes, RCRI value 2. Age, renal impairment, dementia or AF, red blood cell transfusions, new ECG changes, RCRI value
Ma, 2015 [31]	30 days	0/2519	MACE (cardiac death, non-fatal MI, cardiac arrest)	251/2519 (10)	NR/NR	NR/NR	NR, NR	NR; NR; NR	OR 8.74; 5.881–12.987; <0.001	Age, sex, co-morbidities, preoperative medications
Maile, 2016 [32]	30 days	0^††^/4575	Mortality	281/4575 (6)	112/986	169/3589	40, 80	NR; NR; NR°/OR 2.6; 2.0–3.3; <0.0001°°	NR; NR; NR	NA
Thomas, 2016 [33]	5 days	0^††^/85	Combined myocardial injury (MI and MINS)	39/85 (46)	17/32	22/53^***^	44, 67	‘baseline hs-TnT did predict postoperative cMInj in this sample’°/OR 1.6; 0.7–3.9; 0.3°°	NR; NR; NR	NA
Zimmerman, 2016 [34]	30 days	0^††^/464	Mortality	78/464 (17)	28/82	50/382	36, 86	OR 3.53; 1.66–7.47; 0.002°/OR 3.4; 2.0–5.9; <0.0001°°	OR 2.96; 1.1–7.6; 0.025	Age, sex, morbid obesity, diabetes, smoking, functional dependency, COPD, ascites, CHF, acute renal failure, dialysis dependence, cancer, open wound, steroid use, weight loss, bleeding, sepsis, ASA physical status ≥3
Kopec, 2017 [35]	3 days	2/572	MI	30/570 (5)	21/238	9/332	70, 60	OR 3.58; 1.61–7.97; 0.001°/OR 3.5; 1.6–7.7; 0.002°°	OR 2.26; 0.93–5.83; 0.07	Age, sex, eGFR, preexisting CAD
Association between **perioperative change** in cardiac troponin and adverse outcome										
First author, Year	Length of follow-up	No. lost to follow-up/No. patients^§§^	Adverse outcome	No. events/Sample size^§^ (%)	No. events/No. elevated cTn	No. events/No. non-elevated cTn	Sensitivity, Specificity	Unadjusted OR/HR; 95% CI; p-value	Adjusted OR/HR; 95% CI; p-value	Variables adjusted for in multivariate analysis
Nagele, 2013 [26]	3 years	0^††^/608	Mortality	80/608 (13)	NR/NR	NR/NR	NR, NR	HR 1.58; 0.95–2.64; 0.078	NR; NR; NR	NA
Gillmann, 2014 [29]	30 days	0^††^/455	MACE (MI type I/II, cardiovascular death, any new rise in cTn prompted by clinical suspicion for ACS with cut-offs cTnT>0.05 µg/L and hs-cTnT>50 ng/L)	41/455 (9)	34/117	7/338	83, 80	NR; NR; NR°/OR 19.4; 8.3–45.2; <0.0001°°	‘independently associated’	NR
Devereaux, 2017 [12]	30 days	974/8831^***^	Mortality	94/7857 (1)	71/2741	23/5116	76, 66	NR; NR; NR°/OR 5.9; 3.7–9.4; <0.0001°°	HR 4.53; 2.77–7.39; <0.001	Active cancer, general surgery, urgent/emergent surgery, history of PVD, history of COPD, age, recent high-risk CAD, history of stroke, neurosurgery

ACS = Acute coronary syndrome. AF = Atrial fibrillation. AMI = Acute myocardial infarction. ASA = American Society of Anesthesiologists. BMI = Body mass index. BNP = Brain natriuretic peptide. CAD = Coronary artery disease. CHF = Congestive heart failure. CK-MB = Creatine kinase-Muscle/brain. cMInj = Combined myocardial injury. COPD = Chronic obstructive pulmonary disease. CPR = Cardio-pulmonary resuscitation. CRP = C reactive protein. cTn = Cardiac troponin. cTnI = Cardiac troponin I. cTnT = Cardiac troponin T. ECG = Electrocardiogram. eGFR = Estimated glomerular filtration rate. GCS = Glasgow coma scale. h = Hours. hs-cTnT = High-sensitivity cardiac troponin T. HR = Hazard ratio. ICU = Intensive care unit. IHD = Ischemic heart disease. MACE = Major adverse cardiac event. MI = Myocardial infarction. MINS = Myocardial injury after non-cardiac surgery. NA = Not applicable. No. = Number. NR = Not reported. NYHA = New York Heart Association. OR = Odds ratio. PVD = Peripheral vascular disease. RCRI = Revised Cardiac Risk Index. URL = 99th percentile upper reference limit decided by assay manufacturer. VF = Ventricular fibrillation.

§ = Patients included in eligible effect measure analyses.

§§ = Patients at study baseline.

† = Not explicitly stated, calculated by authors.

†† = Not explicitly stated, concluded by authors.

*** = Discrepancy between reported figures at different locations in the article.

Δ = Change.

° = Data reported in study.

°° = Figures used in the meta-analyses, yielded from reconstructing 2x2 contingency tables.

#### Ability of preoperative cardiac troponin to predict short-term adverse outcome—adjusted analysis

**Fig 3:**
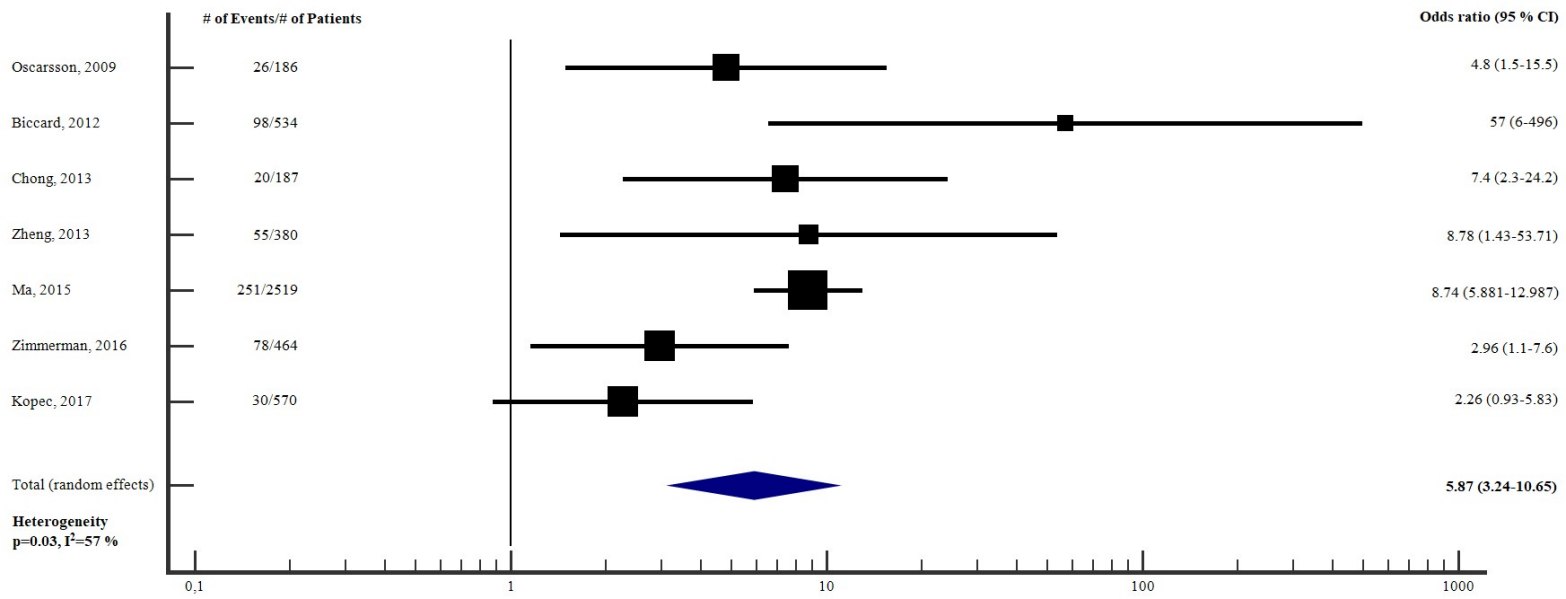
Adjusted odds ratios for elevation in preoperative cTn to predict short-term adverse outcome. Forest plot showing the individual and pooled adjusted odds ratios from the included studies. cTn = Cardiac troponin. CI = Confidence interval. # = Number of.

Fig 3 reports the meta-analysis of the seven studies [19, 23, 25, 28, 31, 34, 35] for which we could obtain an aOR for preoperative cTn to predict short-term adverse outcome. The total sample size was 4840 (range 186 to 2519). Preoperative cTn was an independent predictor of short-term adverse outcome (aOR 5.87, 95% CI 3.24–10.65, p<0.001), however there was substantial heterogeneity between studies (I^2^ = 57%, p = 0.03). There was great variability with respect to number and type of variables adjusted for. All studies adjusted for cardiovascular comorbidity, however only two of the studies [19, 23] adjusted for RCRI. (Table 3). Subgroup meta-analysis, according to the type of troponin assay showed that conventional cTn was clearly predictive of short-term adverse outcome (S3 Fig). It was not possible to conduct subgroup meta-analysis for hs-cTnT since only one study [35] used this type of assay.

#### Ability of preoperative cardiac troponin to predict long-term adverse outcome—unadjusted analysis

**Fig 4:**
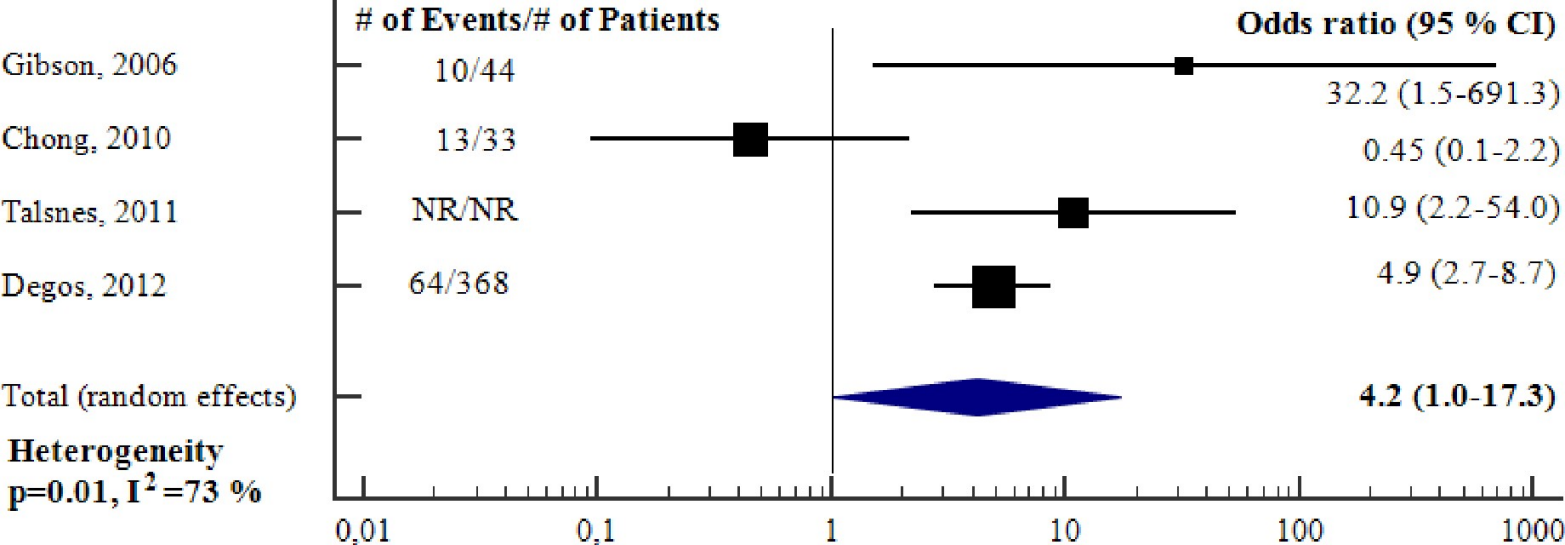
Unadjusted odds ratios for elevation in preoperative cTn to predict long-term adverse outcome. Forest plot showing the individual and pooled unadjusted odds ratios from the included studies. cTn = Cardiac troponin. CI = Confidence interval. # = Number of.

The proportion of patients who had long-term adverse outcomes varied from 13 to 39 per cent. The sensitivity and specificity for preoperative cTn to predict long-term adverse outcome ranged from 23 to 48 and 60 to 100 per cent, respectively. (Table 3). Fig 4 reports the meta-analysis of the four studies [18, 20, 21, 24] for which we could obtain an OR for preoperative cTn to predict long-term adverse outcome. The total sample size was less than 600 (one study [21] did not report sample size). Preoperative cTn was a significant, unadjusted predictor of long-term adverse outcome (OR 4.2, 95% CI 1.0–17.3, p = 0.05), however there was substantial heterogeneity between studies (I^2^ = 73%, p = 0.01). (Table 3). All four studies used conventional cTn, consequently it was not applicable to conduct subgroup meta-analyses according to the type of troponin assay.

#### Ability of preoperative cardiac troponin to predict long-term adverse outcome—adjusted analysis

**Fig 5:**
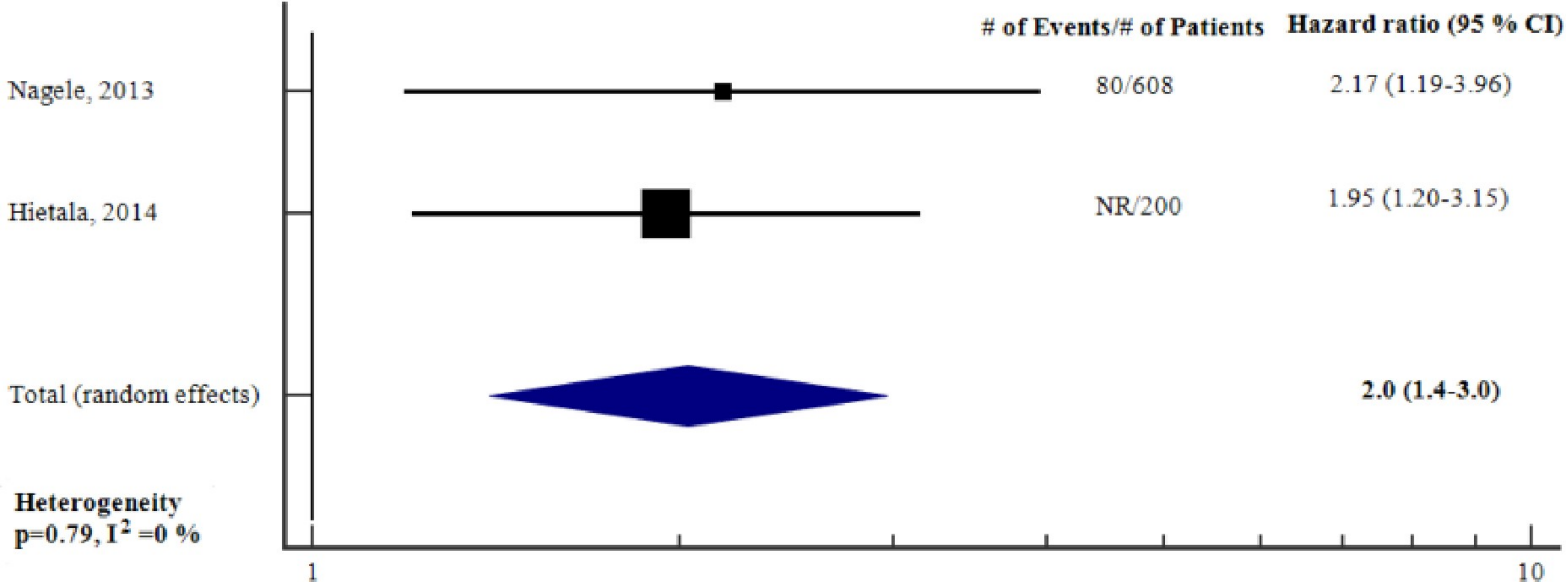
Adjusted hazard ratios for elevation in preoperative cTn to predict long-term adverse outcome. Forest plot showing the individual and pooled adjusted hazard ratios from the included studies. cTn = Cardiac troponin. CI = Confidence interval. # = Number of.

Fig 5 reports the meta-analysis of the two studies [26, 30] for which we could obtain an aHR for preoperative cTn to predict long-term adverse outcome. The total sample size was 808. Preoperative cTn was an independent predictor of long-term adverse outcome (adjusted HR 2.0, 95% CI 1.4–3.0, p<0.001), heterogeneity was low (I2 = 0%, p = 0.79). There was variability with respect to type of variables adjusted for. Both studies adjusted for cardiovascular comorbidity, however only one of the studies [30] adjusted for RCRI. (Table 3). As there were only two studies investigating the association, it was not possible to conduct further subgroup meta-analyses according to the type of troponin assay.

### Perioperative change in cardiac troponin

#### Study and patient related characteristics

Three studies with a total sample size was 8920 (range 455 to 7857) reported data for changes in cTn. All three studies [12, 26, 29] were prospective cohort studies, one of them performed as a large, international multicenter study [12]. The studies included patients undergoing a wide range of surgical procedures with varying risk. (Table 2).

#### Cardiac troponin related characteristics

All three studies [12, 26, 29] evaluated changes in hs-cTnT (i.e. change between pre- and postoperative cTn). The cut-off for absolute change in hs-cTnT ranged from 6.3 ng/L to 14 ng/L. Most of the preoperative blood sampling was conducted on the day of surgery. The postoperative blood was sampled serially until day three after surgery in two of the studies [12, 26], whereas one study [29] sampled blood only once, 24 hours after surgery. (S2 Table).

#### Ability of perioperative change in cardiac troponin to predict short-term adverse outcome—unadjusted analysis

**Fig 6:**
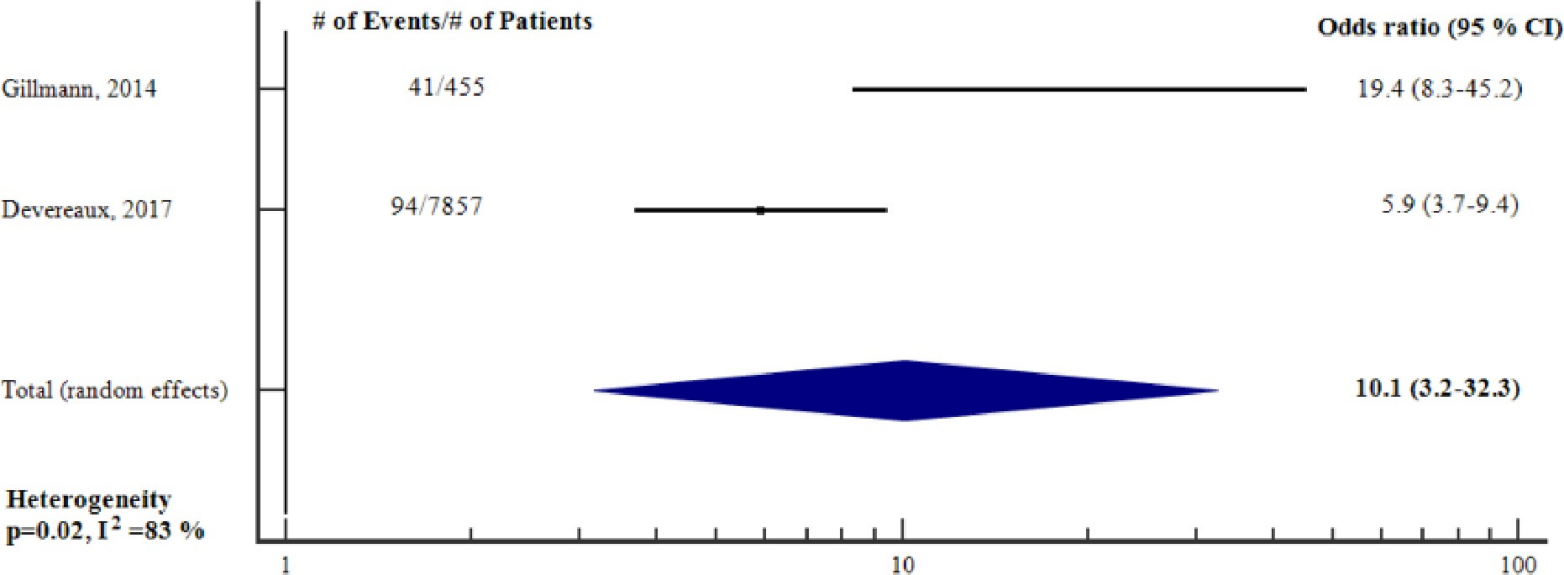
Unadjusted odds ratios for an absolute perioperative change in cTn to predict short-term adverse outcome. Forest plot showing the individual and pooled unadjusted odds ratios from the included studies. cTn = Cardiac troponin. CI = Confidence interval. # = Number of.

The proportion of patients who had short-term adverse outcome varied from 1 to 9 per cent. The sensitivity and specificity for a change in hs-cTnT to predict short-term adverse outcome ranged from 76 to 83 and 66 to 80 per cent, respectively. Fig 6 reports the meta-analysis of the two studies [12, 29] for which we could obtain an unadjusted OR for an absolute change in hs-cTnT to predict short-term adverse outcome. The total sample size was 8312. An absolute change in hs-cTnT was a significant, unadjusted predictor of short-term adverse outcome (unadjusted OR 10.1, 95% CI 3.2–32.3, p<0.001), however there was a substantial heterogeneity (I^2^ = 83%, p = 0.02).

#### Ability of perioperative change in cardiac troponin to predict short-term adverse outcome—adjusted analysis

Devereux et al. [12] demonstrated that an absolute change in hs-cTnT was an independent predictor (aHR 4.53, 95% CI 2.77–7.39, p <0.001) of short-term adverse outcome. The authors adjusted for preoperative and surgical variables, previously associated with 30-day mortality [46]. In addition, Gillmann et al. [29] stated that an absolute change in hs-cTnT was independently associated with short-term adverse outcome, however the actual effect size was not reported. (Table 3).

#### Ability of perioperative change in cardiac troponin to predict long-term adverse outcome—unadjusted analysis

Only one study reported the association between change in cTn and long-term adverse outcome [26]. An absolute change in hs-cTnT was associated with long-term adverse outcome (HR 1.58 (95% CI, 0.95–2.64, p = 0.078). There were no studies reporting adjusted effect sizes for long-term adverse outcome. (Table 3).

#### Prognostic performance of preoperative cardiac troponin to predict short-term adverse outcome

**Fig 7:**
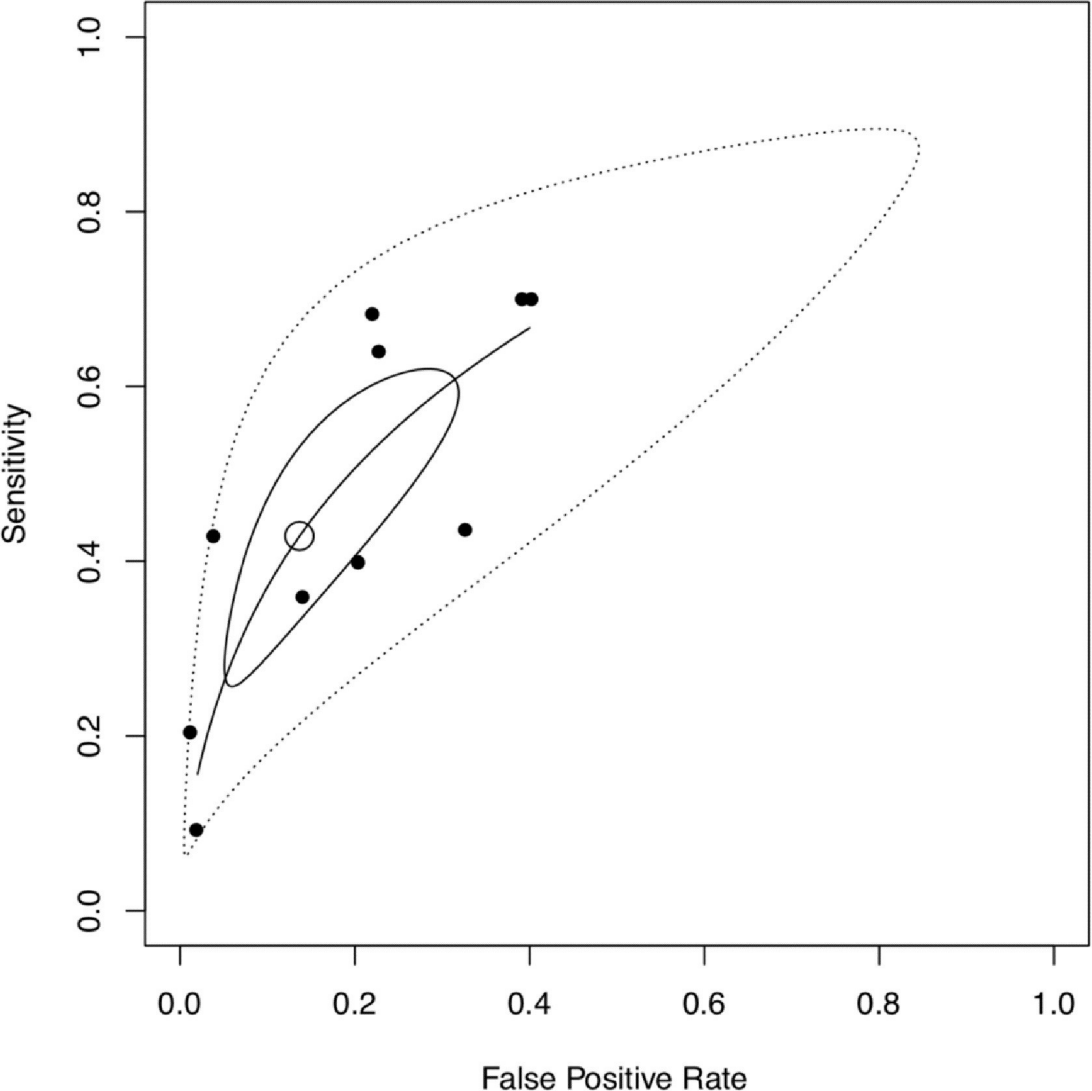
Summary receiver operating characteristic (SROC) curve for prognostic performance of preoperative cTn to predict short-term adverse outcome. Filled dots = observed data. Unfilled dot = pooled sensitivity (0.43 [CI 0.29–0.58]) and specificity (0.85 [CI 0.73–0.94]). Black curve = SROC curve (AUC = 0.68). Black-lined region = 95% confidence region. Dashed-lined region = 95% prediction region.

A forest plot of the sensitivities and specificities for preoperative cTn to predict short-term adverse outcome [17, 23, 26–29, 32–35] demonstrated significant heterogeneity (χ^2^ = 80.1, p<0.001 and χ^2^ = 723.7, p<0.001 respectively) between studies. (S4 Fig). A summary ROC curve returned an AUC of 0.68 with a large prediction region. The pooled sensitivities and specificities were 0.43 (CI 0.29–0.58) and 0.85 (CI 0.73–0.94) respectively. (Fig 7).

There were only three studies of preoperative cTn and long-term adverse outcome [18, 20, 24], and only two studies of changes in cTn and any adverse outcome [12, 29]. Forest plots of sensitivity and specificities and SROC curves were therefore not generated for these studies.

## Discussion

This meta-analysis suggests that preoperative cTn predicts adverse outcome defined as MACE and/or all-cause mortality in adult noncardiac surgical patients. The effect was best demonstrated for short-term adverse outcome, and was sustained in adjusted analyses. In addition, pooled estimates show that cTn predicts adverse outcome, regardless of follow-up time, adjustment for confounders, and whether cTn was considered as preoperative values or perioperative changes. Additional subgroup meta-analysis, according to the type of troponin assay showed that preoperative cTn was clearly predictive of short-term adverse outcome in both subgroups of studies (i.e. conventional cTnT/cTnT and hs-cTnT). This is in line with the recent study by Gualandro et al. [47]. Despite this demonstrated effect, analysis of the prognostic performance of preoperative cTns was poor for short-term adverse outcome. Thus, although this meta-analysis suggests that preoperative cTn predicts adverse outcome, SROC analysis reveals that the prognostic performance of preoperative cTn is poor. For long-term adverse outcome and perioperative change in cTn we were unable to adequately evaluate the prognostic performance.

Increased postoperative cTn is associated with increased risk of adverse events and guidelines now suggest postoperative surveillance for myocardial injury in high-risk patients [6, 7, 9]. The present study indicates that this might also apply to preoperative measurements although the evidence is less strong. Our findings support the recent meta-analysis by Shen et al. [15] demonstrating independent associations between increased preoperative cTn and short-term MACE and mortality. We add to those findings by including studies for long-term adverse outcome, where unadjusted and adjusted analyses demonstrated a relationship with cTn.

Since many patients have elevations in preoperative cTn, we also investigated the predictive value of perioperative changes. This is relevant since perioperative changes in cTns may imply differences in outcomes and perioperative management. Although this meta-analysis indicates that a perioperative change in cTn might have a prognostic value for short-term adverse outcome after noncardiac surgery, these were unadjusted estimates and we were not able to perform a meta-analysis with a change in cTn as adjusted predictor. Furthermore, there was substantial heterogeneity and the findings were based on only two studies. Thus, were we unable to support or refute the findings of Gillmann et al. [29] where perioperative changes in cTn improved the reclassification of patients with non-events. Lastly, a change in cTn might have a prognostic value for long-term adverse outcome, however we were only able to identify one, relatively small study investigating this association. In a recent study not included within the time frame for our meta-analysis, Puelacher et al. [48] showed that an absolute change in perioperative cTn of ≥ 14 ng/L was significantly associated with both short- and long-term adverse outcome after noncardiac surgery, even after adjustment for other confounders.

Although the meta-analysis demonstrated the association between cTn and adverse outcome, these were derived from multivariable analyses that only variably adjusted for relevant confounders. To determine whether predictors identified in multivariable analyses significantly improve preoperative risk prediction compared to current risk scores, the net reclassification index (NRI) may be used. The net reclassification index objectively evaluates the prognostic performance of a risk predictor when added on to current risk prediction methods, calculated as the difference between the proportion of patients correctly and incorrectly reclassified according to the study outcome. We identified only three studies reporting the NRI. Generally, an improvement in NRI was seen using preoperative [23, 26, 29] or perioperative changes in cTns [29]. However, a potential for misclassification was also demonstrated by misclassifying patients with events into a low-risk group [23, 29]. Regarding the prognostic performance of preoperative cTn for short-term adverse outcome, our results indicate that it has only moderate predictive ability (AUC for SROC curve 0.68). Importantly the lower boundary of the 95% prediction region approaching the line of equality, indicating that test performance is poor.

### Strengths and limitations

Strengths of our study include conducting and reporting the study in accordance with the PRISMA statement [16], the comprehensive and reproducible search strategy including reviewing relevant reference lists and lastly conducting eligibility decisions, data extraction and bias assessment in duplicate. We recognize that our study has several limitations. Our eligibility criteria were liberal to ensure inclusion of studies in a heterogeneous field. This is reflected in the heterogeneity across the studies, complicating the interpretation of our findings. On the other hand, it is our appraisal that with stricter eligibility criteria we would have excluded several important individual studies, consequently missing out on valuable information. Regarding preoperative cTn, the most important limitation was the substantial heterogeneity across the studies, where important differences exist regarding type of surgery, assay manufacturer, timing of cTn sampling, type of cTn, prognostic cut-off concentration and outcome. There were also significant methodological limitations in the included studies. In the unadjusted meta-analysis for short-term adverse outcome, one study constituting fifty per cent of the total sample size was a retrospective study [32], with a high-risk of selection bias. Forty per cent of the studies adjusted for more than one variable per ten outcome events [19, 34, 35], creating a risk of unreliable estimates [49]. Yet, a majority of the studies did not adjust for important potential confounders, e.g. the RCRI, reducing the reliability in assessing the independent prognostic value of preoperative cTn. For perioperative changes in cTn, the most essential limitation was that the findings were based on one study only [12], although we acknowledge that this was a rigorously conducted multicenter international study. Moreover, perioperative changes were only considered for a subgroup of patients. The recently published study by Puelacher et al. [48] however supports the finding by Devereaux et al. [12] that perioperative change in cTn is associated with short-term adverse outcome.

The relationship between sensitivity and specificity is essential in evaluating the prognostic performance of a test. In our study, since different criteria (e.g. type of troponin assay, cut-offs, patient populations) have been used, there will be different relationships between sensitivity and specificity across the studies. As sensitivity increases, specificity will generally drop (threshold effect). Thus, averaging sensitivities and specificities across may not reflect the overall accuracy of the test, and extremes of threshold criteria can cause inaccurate interpretations of the pooled results. We used the SROC curve as way of overcoming this problem. Our study demonstrated only an ‘acceptable’ AUC with a large prediction region indicating a heterogeneity in the underlying studies. However, there are also limitations relating to the SROC analysis itself. For example, although the estimates are weighted, there is a bias towards tests with lower diagnostic accuracy. Further, primary studies are assumed to be random samples of the larger overall ‘SROC population’, and that differences in results are due to random error [50].

Regarding applicability of our findings, it should be taken into account that only one [12] of the included studies fully covered a broad spectrum of ‘adult patients undergoing non- cardiac surgery’ when considering the following as ‘broad spectrum’: wide range of age; equal sex proportion; wide range of non-cardiac surgery; all risks of surgery; both elective and emergent surgery and no specific risk factors or comorbidities in inclusion criteria. The applicability is especially relevant for long-term outcome, since these findings were based on few studies.

### Implications for research and practice

Is preoperative cTn a good screening tool for increased mortality and MACE after noncardiac surgery? Although conventional meta-analyses of risk estimates support the prognostic value of preoperative, and to a lesser extent, perioperative changes in cTn, a meta-analysis of the sensitivities and specificities for preoperative cTn suggest that prognostic performance is still inadequate. However, the summary statistic was limited by differences in assays, cutoffs, study populations and outcomes, limiting their interpretation. To potentially improve our understanding, we suggest that future studies use the current gold standard for assays, hs-cTn. We also suggest measurement of both pre- and postoperative levels [11], with evaluation of confounding factors to help elucidate the meaning of pre- and perioperative changes in troponin levels. Furthermore, we suggest investigation of whether perioperative hs-cTn alone or in a panel of other risk factors (e.g. other biomarkers and clinical factors) provides better risk stratification compared to the current golden standard [2, 5]. In this regard, the net classification index that attempts to quantify how well a new model reclassifies subjects to the correct group may be a useful analysis. This is the subject of an ongoing study (NCT03436238). If the prognostic value of preoperative cTn and perioperative changes in cTns are confirmed, targeted interventions based on cTn should be evaluated in high-quality, randomized controlled trials. To our knowledge, there are currently no such published studies. A knowledge of the mechanism behind poor postoperative outcomes would help in designing these studies and is the subject of one ongoing prospective trial (NCT03317561).

### Conclusion

Our study indicates that although preoperative cTn and perioperative change in cTn might be valuable predictors of MACE and/or all-cause mortality in adult noncardiac surgical patients, its overall prognostic performance remains uncertain. Future large, representative, high-quality studies are needed to establish the potential role of cTns in perioperative cardiac risk stratification.

## Supporting information

S1 File Full search strategy. (DOCX)

S2 File Prospero protocol. (PDF)

S3 File Key characteristics and variables for QUIPS tool. (DOCX)

S1 Table 2x2 contingency tables/calculation of sensitivity & specificity. (DOCX)

S2 Table Cardiac troponin related characteristics. (DOCX)

S1 Fig Unadjusted odds ratios for elevation in preoperative conventional cTn to predict short-term adverse outcome. Forest plot showing the individual and pooled unadjusted odds ratios from the included studies. cTn = Cardiac troponin. CI = Confidence interval. # = Number of.
(TIF)

S2 Fig Unadjusted odds ratios for elevation in preoperative hs-cTnT to predict short-term adverse outcome. Forest plot showing the individual and pooled unadjusted odds ratios from the included studies. cTn = Cardiac troponin. CI = Confidence interval. # = Number of.
(TIF)

S3 Fig Adjusted odds ratios for elevation in preoperative conventional cTn to predict short-term adverse outcome. Forest plot showing the individual and pooled adjusted odds ratios from the included studies. cTn = Cardiac troponin. CI = Confidence interval. # = Number of.
(TIF)

S4 Fig Forest plot (sensitivity & specificity): Preoperative cTn and short-term outcome. (TIF)

S4 File Prisma checklist. (DOC)
