# Supplementary material for: Prognostic performance of preoperative cardiac troponin and perioperative changes in cardiac troponin for the prediction of major adverse cardiac events and mortality in noncardiac surgery: A systematic review and meta-analysis
Source: PLoS One. 2019 Apr 22;14(4):e0215094. doi: 10.1371/journal.pone.0215094 (PMC6476502; doi:10.1371/journal.pone.0215094)
Supplement: S1 File — (DOCX) [file pone.0215094.s001.docx]

S1 File – Full search strategy

Searches were conducted on the 23^rd^ of January 2016 and again on the 23^rd^ of June 2017. No filters with respect to year of publication or language were used.

MEDLINE (1966 to 23^rd^ of January 2016 and 23^rd^ of January 2016 to 23^rd^ of June 2017) and OLDMEDLINE (1940^th^ to 23^rd^ of January 2016 and 23^rd^ of January 2016 to 23^rd^ of June 2017) via PubMed

1. Troponin[Mesh]

2. troponin

3. 1 OR 2

4. Surgical Procedures, Operative[Mesh] OR surgery[Subheading] OR Postoperative

Complications[Mesh]

5. surgery

6. 4 OR 5

7. Cardiovascular Diseases[Mesh] OR Mortality[Mesh] OR Death[Mesh]

8. "heart arrest" OR "cardiac arrest" OR "myocardial infarction" OR "heart failure" OR

"cardiac events" OR "cardiac arrhythmia" OR angina OR mortality OR death OR "fatal outcome"

9. 7 OR 8

10. Prognosis[Mesh] OR Risk Assessment[Mesh] OR Sensitivity and Specificity[Mesh]

11. prognosis OR prognostic OR predict* OR "risk assessment"

12. 10 OR 11

13. Perioperative Care[Mesh] OR Perioperative Period[Mesh]

14. perioperative OR preoperative OR postoperative

15. 13 OR 14

16. 3 AND 6 AND 9 AND 12 AND 15

EMBASE (1974 to 23^rd^ of January 2016 and 23^rd^ of January 2016 to 23^rd^ of June 2017) via Ovid

1. exp troponin/

2. troponin.mp.

3. 1 OR 2

4. exp surgery/

5. surgery.mp.

6. 4 OR 5

7. exp cardiovascular disease/

8. exp mortality/

9. exp death/

10. cardiovascular disease.mp.

11. mortality.mp.

12. death.mp.

13. fatal outcome.mp.

14. 7 OR 8 OR 9 OR 10 OR 11 OR 12 OR 13

15. exp “prediction and forecasting”/

16. exp risk assessment/

17. prognostic.mp.

18. predict*.mp.

19. risk assessment.mp.

20. 15 OR 16 OR 17 OR 18 OR 19

21. exp perioperative period/

22. exp preoperative period/

23. exp postoperative period/

24. perioperative.mp.

25. preoperative.mp.

26. postoperative.mp.

27. 21 OR 22 OR 23 OR 24 OR 25 OR 26

28. 3 AND 6 AND 14 AND 20 AND 27
