# Supplementary material for: Prognostic performance of preoperative cardiac troponin and perioperative changes in cardiac troponin for the prediction of major adverse cardiac events and mortality in noncardiac surgery: A systematic review and meta-analysis
Source: PLoS One. 2019 Apr 22;14(4):e0215094. doi: 10.1371/journal.pone.0215094 (PMC6476502; doi:10.1371/journal.pone.0215094)
Supplement: S2 File — (PDF) [file pone.0215094.s002.pdf]

The prognostic value of preoperative and perioperative changes in cardiac troponins for the prediction of major adverse cardiac events and mortality in patients undergoing non-cardiac surgery

Michelle Chew, Caroline Humble, Ib Jammer

Citation

Michelle Chew, Caroline Humble, Ib Jammer. The prognostic value of preoperative and perioperative changes in cardiac troponins for the prediction of major adverse cardiac events and mortality in patients undergoing non-cardiac surgery. PROSPERO 2018 CRD42018094773 Available from: http://www.crd.york.ac.uk/PROSPERO/display_record.php?ID=CRD42018094773

Review question

1) Is preoperative cardiac troponin (cTn) a predictor of death and/or major adverse cardiac events (MACE)?
2) Are perioperative changes in cardiac troponin (cTn) a predictor of death and/or major adverse cardiac events (MACE)?

Searches

We conducted two searches in the electronic databases, MEDLINE via PubMed and Embase via Ovid.
No filters with respect to year of publication or language were used.
The first search was conducted on 23rd Jan 2016, and the second search was an update extending to 23rd June 2017.

The reference lists of the included studies and reviews were also hand searched.
The following MeSH terms were used in the MEDLINE search: 'Troponin', 'Surgical Procedures, Operative'/'surgery'/'Postoperative Complications', 'Cardiovascular Diseases'/'Mortality'/'Death', 'Prognosis'/'Risk Assessment'/'Sensitivity and Specificity', 'Perioperative Care'/'Perioperative Period'.

Additional search strategy information can be found in the attached PDF document (link provided below).

Types of study to be included

Inclusion criteria:

All study designs will be included.
Human adults ?18 years old.

Patients undergoing non-cardiac surgery.
Studies measuring cTn, pre- and/or pre- and postoperatively, investigating the association between preop cTn and outcome(s) and/or cTn-change (postop cTn compared to preop cTn) and outcome(s).
Studies assessing cTnT, cTnI, high-sensitivity cTnT (hs-cTnT), high-sensitivity cTnI (hs-cTnI) levels.
Studies assessing all-cause mortality rates, or MACE as defined by the original studies.
Studies assessing unadjusted OR; adjusted OR; unadjusted RR; adjusted RR; unadjusted HR; adjusted HR; numbers available to make a 2x2 table; single p-values, if nothing else is given; relevant quotes on association, if not stated in numbers (e.g. the "unadjusted association was not statistically significant")
Studies in which blood sampling is conducted up to 30 days prior to and after surgery.
Exclusion criteria

Non-full text articles, articles which are not full reports, case series, letters, brief reports will not be included regardless of study design.

Condition or domain being studied

Major adverse cardiac events and mortality after non-cardiac surgery.

Participants/population

Human adults (i.e. ?18 years old) undergoing non-cardiac surgery.
Patients undergoing transplantation surgery will be excluded.

Intervention(s), exposure(s)

Pre- and/or pre- and postoperatively measured cardiac troponin concentrations.
The exposure is elevated preoperative cTn concentrations, or changes in cTn concentrations beyond a cut-off level as determined by the original studies.

Comparator(s)/control

The control will be those patients who do not have an elevated cTn concentrations.

Context

Human adults undergoing non-cardiac surgery.

Primary outcome(s)

Any mortality and/or major adverse cardiac events (MACE) at longest follow-up.
MACE will be defined as per the original studies.

Timing and effect measures
Short-term: <= 30 days after surgery or in-hospital.
Long-term: >30 days after surgery.

Secondary outcome(s)

None.

Data extraction (selection and coding)

Two authors will independently screen titles and abstracts in accordance with predefined eligibility criteria. Differing opinions regarding whether to include or exclude full-text articles will be resolved by discussion between the two authors. If consensus cannot be reached, a third author will review the full-text article in question, and will made the final decision. Data extraction (ongoing) will be independently conducted by two authors and any disagreements resolved by discussion.
A predefined data extraction template will be used to collect relevant information from the included studies.
We will extract the following data from all eligible articles:
Baseline data: first author, year of publication, study design, number of participating centers, study period, sample size (i.e. number of patients included in the statistical analysis), type of surgery, risk of surgery, urgency of surgery, mean or median age (if not explicitly stated, we calculated it if possible), male proportion (if not explicitly stated, we calculated it if possible).
Troponin data: cTn type, assay manufacturer, timing and frequency of cTn sampling (i.e. for the cTn included as prognostic factor in the statistical analysis), prognostic cTn cut-off concentration (we converted all units to ?g/L).
Outcome data: length of follow-up for eligible outcome, proportion between number of patients lost to follow-up and number of patients at study baseline, eligible outcome, proportion of sample size with outcome event, proportion of patients with elevated cTn with outcome event, proportion of patients with non-elevated cTn with outcome event, sensitivity and specificity (if not explicitly stated, we will calculate this, if possible), data to construct 2x2 contingency tables, eligible effect measure with 95 % CI and p-value (if reported), variables adjusted for in multivariate analysis (if relevant).

Risk of bias (quality) assessment

We use the Quality In Prognostic Studies (QUIPS) tool to assess the risk of bias in the individual, included studies.
The QUIPS tool consists of six, separate bias domains: selection bias; attrition bias; prognostic factor (i.e. cTn) measurement bias; outcome measurement bias (i.e. eligible outcome); study confounding and finally,

bias related to statistical analysis and reporting. Importantly, for the study confounder assessment we predefined six important, potential confounders: age; RCRI score (in any way it was adjusted for); pre-existing kidney disease or injury; peripheral vascular disease; urgency of surgery and length of surgery. For each of the six domains the risk of bias is assessed as low, moderate or high.
The bias assessment will be conducted independently by two authors, and different opinions regarding any issue will be resolved through discussion between the two authors.

Strategy for data synthesis

We plan to conduct a meta-analysis of the studies if the data are sufficiently homogeneous.
We aim to extract data to construct 2x2 contingency tables using aggregate data and to calculate odds ratios using both the Cochran’s Q statistic and the I^2 statistic. Random effects models will be used to account for the heterogeneity of the included studies, and weighted RR and 95% CI will be calculated for the outcomes.
If the studies are not sufficiently homogeneous, we plan a qualitative synthesis.

Analysis of subgroups or subsets

Preoperative cTn short- and long-term outcomes unadjusted effect size.
Changes in cTn (maximum postoperative minus preoperative values).
Where possible we will also conduct a meta-analysis on adjusted effect sizes.

Contact details for further information

Michelle Chew
michelle.chew@liu.se

Organisational affiliation of the review

Linkoeping University

Review team members and their organisational affiliations

Dr Michelle Chew. Linkoeping University
Dr Caroline Humble.
Dr Ib Jammer. Bergen University Hospital

Collaborators

Dr Jonas Bjork. Lund University
Mr Matthias Bank. Lund University

Anticipated or actual start date

23 June 2017

Anticipated completion date

31 August 2018

Funding sources/sponsors

Region Östergotland County Council

Conflicts of interest

None specified.

Language

English

Country

Sweden

Stage of review

Review_Ongoing

Subject index terms status
Subject indexing assigned by CRD

Subject index terms
Cardiovascular System; Clinical Decision-Making; Death; Heart; Humans; Mortality; Natriuretic Peptide, Brain; Perioperative Period; Postoperative Complications; Preoperative Period; Prognosis; Surgical Procedures, Operative; Treatment Outcome; Troponin

Date of registration in PROSPERO
23 May 2018

Date of publication of this version
23 May 2018

Details of any existing review of the same topic by the same authors

Stage of review at time of this submission

Stage	Started	Completed
Preliminary searches	Yes	Yes
Piloting of the study selection process	Yes	Yes
Formal screening of search results against eligibility criteria	Yes	Yes
Data extraction	Yes	No
Risk of bias (quality) assessment	Yes	No
Data analysis	No	No

Versions
23 May 2018

PROSPERO
This information has been provided by the named contact for this review. CRD has accepted this information in good faith and registered the review in PROSPERO. CRD bears no responsibility or liability for the content of this registration record, any associated files or external websites.
