# Supplementary material for: Prognostic performance of preoperative cardiac troponin and perioperative changes in cardiac troponin for the prediction of major adverse cardiac events and mortality in noncardiac surgery: A systematic review and meta-analysis
Source: PLoS One. 2019 Apr 22;14(4):e0215094. doi: 10.1371/journal.pone.0215094 (PMC6476502; doi:10.1371/journal.pone.0215094)
Supplement: S3 File — (DOCX) [file pone.0215094.s003.docx]

S3 File – Key characteristics and variables for QUIPS tool^38^

| Bias Domain | Key characteristics and variables |
| --- | --- |
| 1.Study Participation  *Source of target population* | - Human adults, i.e. ≥18 years. - Undergoing non-cardiac surgery, excluding transplantation surgery. - Blood sampled for pre- and/or postoperative cardiac troponin. |
| 1.Study Participation  *Baseline characteristics* | - Age - Sex - Revised Cardiac Risk Index - Type of surgery - Urgency of surgery |
| 2.Study Attrition  *Outcome and prognostic factor information on those lost to follow-up* | - Age - Sex - Revised Cardiac Risk Index - Type of surgery - Urgency of surgery - Information on outcome before patient was lost to follow-up |
| 5.Study Confounding  *Important Confounders Measured* | - Age - Revised Cardiac Risk Index score (in any way it was adjusted for) - Pre-existing kidney disease/injury - Peripheral vascular disease - Urgency of surgery - Length of surgery |
