# Supplementary material for: Prognostic performance of preoperative cardiac troponin and perioperative changes in cardiac troponin for the prediction of major adverse cardiac events and mortality in noncardiac surgery: A systematic review and meta-analysis
Source: PLoS One. 2019 Apr 22;14(4):e0215094. doi: 10.1371/journal.pone.0215094 (PMC6476502; doi:10.1371/journal.pone.0215094)
Supplement: S1 Table — (DOCX) [file pone.0215094.s004.docx]

| Münzer, 1996 [17] | 3 day re-MI  (Yes) | 3 day re-MI  (No) |  |
| --- | --- | --- | --- |
| cTnT ≥ 0.2 ng/mL | 2 | 6 | 8 |
| cTnT < 0.2 ng/mL | 4 | 127 | 131 |
| Sensitivity: 33.33 (4.33-77.72)  Specificity: 95.49 (90.44-98.33) | 6 | 133 | 139 |
|  | 3 day left ventricular failure | No 3 day left ventricular failure |  |
| cTnT ≥ 0.2 ng/mL | 3 | 5 | 8 |
| cTnT < 0.2 ng/mL | 4 | 127 | 131 |
| **Total** | 7 | 132 | 139 |
| Sensitivity: 42.86 % (9.90-81.59)  Specificity: 96.21 % (91.38-98.76) |  |  |  |

S1 Table – 2x2 contingency tables/calculation of sensitivity & specificity

| Gibson, 2006 [18] | 6 week cardiac event  (Yes) | 6 week cardiac event  (No) |  |
| --- | --- | --- | --- |
| ’Rise in preoperative cTnI’ (Yes) | 3 | 0 | 3 |
| ‘Rise in preoperative cTnI’ (No) | 7 | 34 | 41 |
| **Total** | 10 | 34 | 44 |
| Sensitivity: 30.00 % (6.67-65.25)  Specificity: 100.00 % (89.72-100.00) |  |  |  |

| Oscarsson, 2009 [19] | 30 day MACE  (Yes) | 30 day MACE  (No) |  |
| --- | --- | --- | --- |
| cTnI >0.06 µg/L | ? | ? | 40 |
| cTnI ≤0.06 µg/L | ? | ? | 146 |
|  | 26 | 160 | 186 |
| Sensitivity: ?  Specificity: ? |  |  |  |
|  | 30 day mortality  (Yes) | 30 day mortality  (No) |  |
| cTnI >0.06 µg/L | ? | ? | 40 |
| cTnI ≤0.06 µg/L | ? | ? | 146 |
| **Total** | 23 | 165 | 186 |
| Sensitivity: ?  Specificity: ? |  |  |  |
|  | 3 month mortality  (Yes) | 3 month mortality  (No) |  |
| cTnI >0.06 µg/L | ? | ? | 40 |
| cTnI ≤0.06 µg/L | ? | ? | 146 |
| **Total** | 43 | 143 | 186 |
| Sensitivity: ?  Specificity: ? |  |  |  |

| Chong, 2010 [20] | 6 month mortality  (Yes) | 6 month mortality  (No) |  |
| --- | --- | --- | --- |
| cTnI >0.05 µg/L | 3 | 8 | 11 |
| cTnI ≤0.05 µg/L | 10 | 12 | 22 |
| Total | 13 | 20 | 33 |
| Sensitivity: 23.08 % (5.04-53.81)  Specificity: 60.00 % (36.05-80.08) |  |  |  |

| Talsnes, 2011 [21] | 3 month mortality  (Yes) | 3 month mortality  (No) |  |
| --- | --- | --- | --- |
| cTnT >0.04 µg/L | ? | ? |  |
| cTnT ≤0.04 µg/L | ? | ? |  |
| Total | ? | ? | 146 |
| Sensitivity: ?  Specificity: ? |  |  |  |

| Alcock, 2012 [22] | In-hospital myocardial necrosis  (Yes) | In-hospital myocardial necrosis  (No) |  |
| --- | --- | --- | --- |
| hs-cTnT ≥14 ng/L | ? | ? | 109 |
| hs-cTnT <14 ng/L | ? | ? | 143 |
| **Total** | 79 | 273 | 352 |
| Sensitivity: ?  Specificity: ? |  |  |  |

| Biccard, 2012 [23] | 30 day MACE  (Yes) | 30 day MACE  (No) |  |
| --- | --- | --- | --- |
| cTnT/cTnI >0.1 ng/mL | 20 | 5 | 25 |
| cTnT/cTnI ≤0.1 ng/mL | 78 | 431 | 509 |
| **Total** | 98 | 436 | 534 |
| Sensitivity: 20.41 % (12.93-29.74)  Specificity: 98.85 % (97.34-99.63) |  |  |  |

| Degos 2012 [24] | 1 year mortality  (Yes) | 1 year mortality  (No) |  |
| --- | --- | --- | --- |
| cTnI >5 µg/L | 31 (64*48.4 %) | 49 (304*16.1%) | 80 |
| cTnI ≤5 µg/L | 33 | 255 | 288 |
| **Total** | 64 | 304 | 368 |
| Sensitivity: 48.44 % (35.75-61.27)  Specificity: 83.88 % (79.26-87.83) |  |  |  |

| Chong, 2013 [25] | In-hospital cardiac event  (Yes) | In-hospital cardiac event  (No) |  |
| --- | --- | --- | --- |
| cTnI ≥5 µg/L | ? | ? | 29 |
| cTnI <5 µg/L | ? | ? | 158 |
| **Total** | 20 | 167 | 187 |
| Sensitivity: ?  Specificity: ) |  |  |  |

| **Preoperative hs-cTnT** | | | |
| --- | --- | --- | --- |
| Nagele, 2013 [26] | 72 h AMI  (Yes) | 72 h AMI  (No) |  |
| hs-cTnT >14 ng/L | 21 (247*8.6 %) | 226 | 247 |
| hs-cTnT <14 ng/L | 9 (361*2.5 %) | 352 | 361 |
| **Total** | 30 | 578 | 608 |
| Sensitivity: 70.00 % (50.60-85.27)  Specificity: 60.90 % (56.79-64.90) |  |  |  |
|  | 3 year mortality  (Yes) | 3 year mortality  (No) |  |
| hs-cTnT >14 ng/L | ? | ? | 247 |
| hs-cTnT <14 ng/L | ? | ? | 361 |
| **Total** | 80 | 528 | 608 |
| Sensitivity: ?  Specificity: ? |  |  |  |
| **Change in hs-cTnT** | | | |
|  | 3 year mortality  (Yes) | 3 year mortality  (No) |  |
| ∆_abs_ hs-cTnT ≥+9 ng/L | ? | ? | ? |
| ∆_abs_ hs-cTnT <+9 ng/L | ? | ? | ? |
| **Total** | 80 | 528 | 608 |
| Sensitivity: ?  Specificity: ? |  |  |  |

| Weber, 2013 [27] | In-hospital mortality  (Yes) | In-hospital mortality  (No) |  |
| --- | --- | --- | --- |
| hsTnT >14 ng/L | 16 | 217 | 233 |
| hsTnT ≤14 ng/L | 9 | 737 | 746 |
| **Total** | 25 | 954 | 979 |
| Sensitivity: 64.00 % (42.52-82.03)  Specificity: 77.25 % (74.46-79.88) |  |  |  |
|  | In-hospital combined endpoint  (Yes) | In-hospital combined endpoint  (No) |  |
| hsTnT >14 ng/L | ? | ? | 233 |
| hsTnT ≤14 ng/L | ? | ? | 746 |
| **Total** | 36 | 943 | 979 |
| Sensitivity: ?  Specificity: ? |  |  |  |

| Zheng, 2013 [28] | In-hospital adverse cardiac event  (Yes) | In-hospital adverse cardiac event  (No) |  |
| --- | --- | --- | --- |
| ‘Positive baseline cTnI’ (Yes) | 5 | 6 | 11 |
| ‘Positive baseline cTnI’ (No) | 49 | 320 | 369 |
| **Total** | 54 | 326 | 380 |
| Sensitivity: 9.26 % (3.08-20.30)  Specificty: 98.16 % (96.04-99.32) |  |  |  |

| **Preoperative hs-cTnT** | | | |
| --- | --- | --- | --- |
| Gillmann, 2014 [29] | 30 day MACE  (Yes) | 30 day MACE  (No) |  |
| hs-cTnT ≥17.8 ng/L | 28 (table 5) | 91 | 119 |
| hs-cTnT <17.8 ng/L | 13 (table 5) | 323 | 336 |
| **Total** | 41 | 414 | 455 |
| Sensitivity: 68.29 % (51.91-81.92)  Specificity: 78.02 % (73.72-81.92) |  |  |  |
| **Change in hs-cTnT** | | | |
|  | 30 day MACE  (Yes) | 30 day MACE  (No) |  |
| ∆_abs_ hs-cTnT ≥6.3 ng/L | 34 | 83 | 117 |
| ∆_abs_ hs-cTnT <6.3 ng/L | 7 | 331 | 338 |
| **Total** | 41 | 414 | 455 |
| Sensitivity: 82.93 % (67.94-92.85)  Specificity: 79.95 % (75.77-83.70) |  |  |  |

| Hietala, 2014 [30] | 30 day mortality  (Yes) | 30 day mortality  (No) |  |
| --- | --- | --- | --- |
| cTnT >0.03 µg/L | ? | ? | 36 |
| cTnT ≤0.03 µg/L | ? | ? | 160 |
| **Total** | 18 (196*9 %) | 178 | 196 |
| Sensitivity: ?  Specificity: ? |  |  |  |
|  | 1000 day mortality  (Yes) | 1000 day mortality  (No) |  |
| cTnT >0.03 µg/L | ? | ? | 36 |
| cTnT ≤0.03 µg/L | ? | ? | 160 |
| **Total** | ? | ? | 196 |
| Sensitivity: ?  Specificity: ? |  |  |  |

| Ma, 2015 [31] | 30 day MACE  (Yes) | 30 day MACE  (No) |  |
| --- | --- | --- | --- |
| cTnI ≥0.07 ng/mL | ? | ? | ? |
| cTnT <0.07 ng/mL | ? | ? | ? |
| **Total** | 251 | 2268 | 2519 |
| Sensitivity: ?  Specificity: ? |  |  |  |

| Maile, 2016 [32] | 30 day mortality  (Yes) | 30 day mortality  (No) |  |
| --- | --- | --- | --- |
| cTnI > 0.10 ng/mL | 112(11.4%) | 874 | 986(21.6%) |
| cTnI ≤ 0.10 ng/mL | 169 (4.7%) | 3420 | 3589 |
| **Total** | 281(6.1%) | 4294 | 4575 |
| Sensitivity: 39.86% (34.04-45.84)  Specificity: 79.65% (78.41-80.84) |  |  |  |

| Thomas, 2016 [33] | 5 day combined myocardial injury  (Yes) | 5 day combined myocardial injury  (No) |  |
| --- | --- | --- | --- |
| hs-cTnT ≥ 14 ng/L | 17 | 15 | 32 |
| hs-cTnT < 14 ng/L | 22 | 31 | 53 |
| **Total** | 39 | 46 | 85 |
| Sensitivity: 43.59 % (27.81-60.38)  Specificity: 67.39 % (51.98-80.47) |  |  |  |

| Zimmerman, 2016 [34] | 30 day mortality  (Yes) | 30 day mortality  (No) |  |
| --- | --- | --- | --- |
| cTnI > 0.15 ng/mL | 28 (82*34 %) | 54 | 82 |
| cTnI ≤ 0.15 ng/mL | 50 (382*13 %) | 332 | 382 |
| **Total** | 78 | 386 | 464 |
| Sensitivity: 35.90 % (25.34-47.56)  Specificity: 86.01 % (82.14-89.31) |  |  |  |

| Devereaux, 2017 [12] | 30 day mortality  (Yes) | 30 day mortality  (No) |  |
| --- | --- | --- | --- |
| hs-cTnT∆_abs_ ≥5 ng/L | 71 | 2670 | 2741 |
| hs-cTnT∆_abs_ <5 ng/L | 23 | 5093 | 5116 |
| **Total** | 94 | 7763 | 7857 |
| Sensitivity: 75.53 % (65.58-83.81)  Specificity: 65.61 % (65.54-66.66) |  |  |  |

| Kopec, 2017 [35] | 5 day MI  (Yes) | 5 day MI  (No) |  |
| --- | --- | --- | --- |
| hs-cTnT > 14 ng/L | 21 | 217 | 238 |
| hs-cTnT < 14 ng/L | 9 | 323 | 332 |
| **Total** | 30 | 540 | 570 |
| Sensitivity: 70.00 % (50.60-85.27)  Specificity: 59.81 % (55.54-63.98 %) |  |  |  |
