# Supplementary material for: Prognostic performance of preoperative cardiac troponin and perioperative changes in cardiac troponin for the prediction of major adverse cardiac events and mortality in noncardiac surgery: A systematic review and meta-analysis
Source: PLoS One. 2019 Apr 22;14(4):e0215094. doi: 10.1371/journal.pone.0215094 (PMC6476502; doi:10.1371/journal.pone.0215094)
Supplement: S2 Table — (DOCX) [file pone.0215094.s005.docx]

| S2 Table. Cardiac troponin related characteristics | | | | |
| --- | --- | --- | --- | --- |
| First author,  Year | Cardiac troponin | Assay manufacturer | Day and frequency of cardiac troponin sampling^§§§^ | Prognostic cut-off concentration of cardiac troponin |
| Münzer,  1996 [17] | cTnT | TnT ELISA (Boehringer, Mannheim, Germany) | Preoperatively: Not further specified | ≥0.2 ng/mL^d^ (0.2 µg/L) |
| Gibson,  2006 [18] | cTnI | ADIVA Centaur®immunoassay (Bayer Diagnostics) | Preoperatively: The evening prior to surgery | ‘a rise in preoperative cTnI’^d^ |
| Oscarsson,  2009 [19] | cTnI | Stratus® CS Acute Care^TM^ Diagnostic System (Dade International Holding GmBH, Lieberbach, Germany) | Preoperatively: Within 1 h before surgery | >0.06 µg/L^d^ |
| Chong,  2010 [20] | cTnI | Beckman-Coulter TnI assay | Preoperatively: Not further specified | >0.05 µg/L^b^ |
| Talsnes,  2011[21] | cTnT | ELICA, Roche, Basel, Switzerland and Abbott, Abbott Park, IL, USA | Preoperatively: 1 day before surgery | >0.04 µg/L^d^  Reference ≤0.01 µg/L |
| Alcock,  2012 [22] | hs-cTnT | Roche Diagnostics, Mannheim, Germany | Preoperatively: In the week preceding surgery | ≥14 ng/L^b^ |
| Biccard,  2012 [23] | cTnT/cTnI (until/from April 2008) | Advia Centaur®Xp (Siemens Healthcare, Malvern, PA, USA) | Preoperatively: Within 24 h before surgery | >0.1 ng/mL^b^ (0.1 µg/L) |
| Degos,  2012 [24] | cTnI | Troponin Ic assay (Stratus Analyzer by Dade, France) | Preoperatively: On admission to ICU | >0.5 µg/L^d^ |
| Chong,  2013 [25] | cTnI | Beckman-Coulter Troponin I assay | Preoperatively: Not further specified | ≥0.05 µg/L^b^ |
| Nagele,  2013 [26] | hs-cTnT | Roche Elecsys 2010 analyzer | Preoperatively: Within 2 h before surgery  Postoperatively: At the end of surgery and on postoperative day 1, 2, 3 | 1. Preoperative >14 ng/L^b^  2. ∆_abs_ ≥+9 ng/L |
| Weber,  2013 [27] | hs-cTnT | Elecsys analyzer (Roche Diagnostics, Mannheim, Germany) | Preoperatively: Within 7 days prior to surgery | >14 ng/L^b^ |
| Zheng,  2013 [28] | cTnI | ACCESS cTnI assay (Beckman Coulter, Inc., Chaska, MN) | Preoperatively: On the morning of surgery | ‘positive baseline cTnI’^d^ |
| Gillmann,  2014 [29] | hs-cTnT | hs-cTnT assay (Roche Diagnostics, Mannheim, Germany) on the Elecsys 2010/cobas e411 immunoanalyzer | Preoperatively: Prior to surgery, not further specified  Postoperatively: 24 h | 1. Preoperative ≥17.8 ng/L^b^  2. ∆_abs_ ≥6.3 ng/L |
| Hietala,  2014 [30] | cTnT | 4^th^ generation TnT assay (ECLIA; Roche Diagnostics GmbH, Mannheim, Germany) | Preoperatively: On admission and before surgery | >0.03 µg/L^d^ |
| Ma,  2015 [31] | cTnI | Dimension Vista 500 Intelligent Laboratory System (Siemens Healthcare Diagnostics, Deerfield, Illinois, United States) | Preoperatively: Upon admission at the hospital (≤24 h before surgery) | ≥0.07 ng/mL^d^ (0.07 µg/L) |
| Maile,  2016 [32] | cTnI | Troponin I Ultra assay; Siemens Healthcare Diagnostics, Deerfield, IL | Preoperatively: Within 30 days before surgery | >0.10 ng/mL^c^ (0.10 µg/L) |
| Thomas,  2016 [33] | hs-cTnT | Roche high-sensitivity assay (Cobas 6000 Analyzer, Roche Diagnostics GmbH, Mannheim, Germany) | Preoperatively: Not further specified | ≥14 ng/L^b^ |
| Zimmerman,  2016 [34] | cTnI | NR | Preoperatively: Not further specified | >0.15 ng/mL^b^ (0.15 µg/L) |
| Devereaux,  2017 [12] | hs-cTnT | Roche 5^th^ generation Elecsys | Preoperatively: Day of surgery (76 %), 1-28 days prior surgery (18 %), prior surgery but ‘date missing’ (6 %)  Postoperatively: 6-12 h, days 1, 2 and 3 | ∆_abs_ ≥5 ng/L |
| Kopec,  2017 [35] | hs-cTnT | Roche Elecsys 2010 analyzer (Roche Diagnostics, Indianapolis, IN) | Preoperatively: Within 2 h before surgery | >14 ng/L^b^ |

cTnI = Cardiac troponin I. cTnT = Cardiac troponin T. hs-cTnT = High-sensitivity cardiac troponin T. h = Hours. URL = 99th percentile upper reference limit decided by assay manufacturer. ICU = Intensive care unit. a = Cut-off = URL. b = Cut-off > URL. c = Cut-off < URL. d = Relationship between cut-off and URL unknown. †† = Not explicitly stated, concluded by authors. §§§ = The timing of the sampling of the eligible cTn. ∆_abs_ = Absolute perioperative change.
