# Supplementary figures and images for: Prognostic performance of preoperative cardiac troponin and perioperative changes in cardiac troponin for the prediction of major adverse cardiac events and mortality in noncardiac surgery: A systematic review and meta-analysis
Source: PLoS One. 2019 Apr 22;14(4):e0215094. doi: 10.1371/journal.pone.0215094 (PMC6476502; doi:10.1371/journal.pone.0215094)

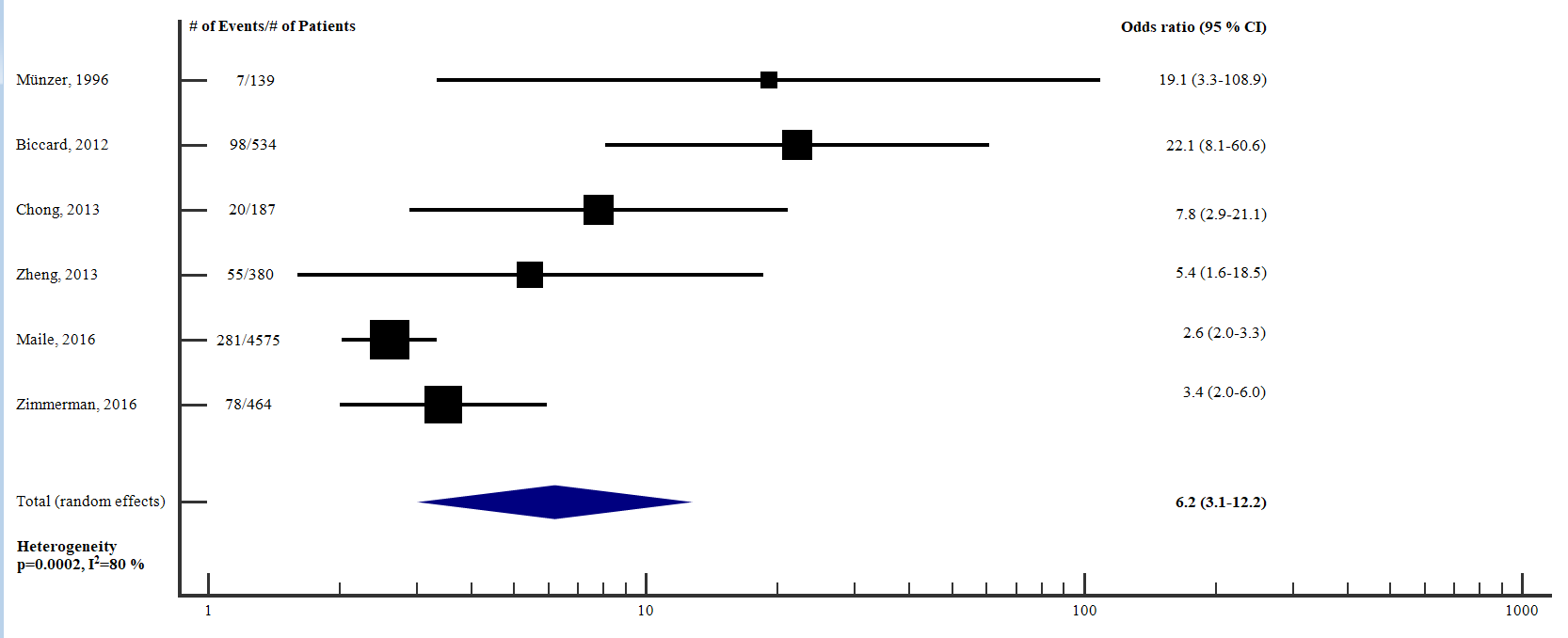

Supplement: S1 Fig — Forest plot showing the individual and pooled unadjusted odds ratios from the included studies. cTn = Cardiac troponin. CI = Confidence interval. # = Number of. (TIF) [file pone.0215094.s006.tif]

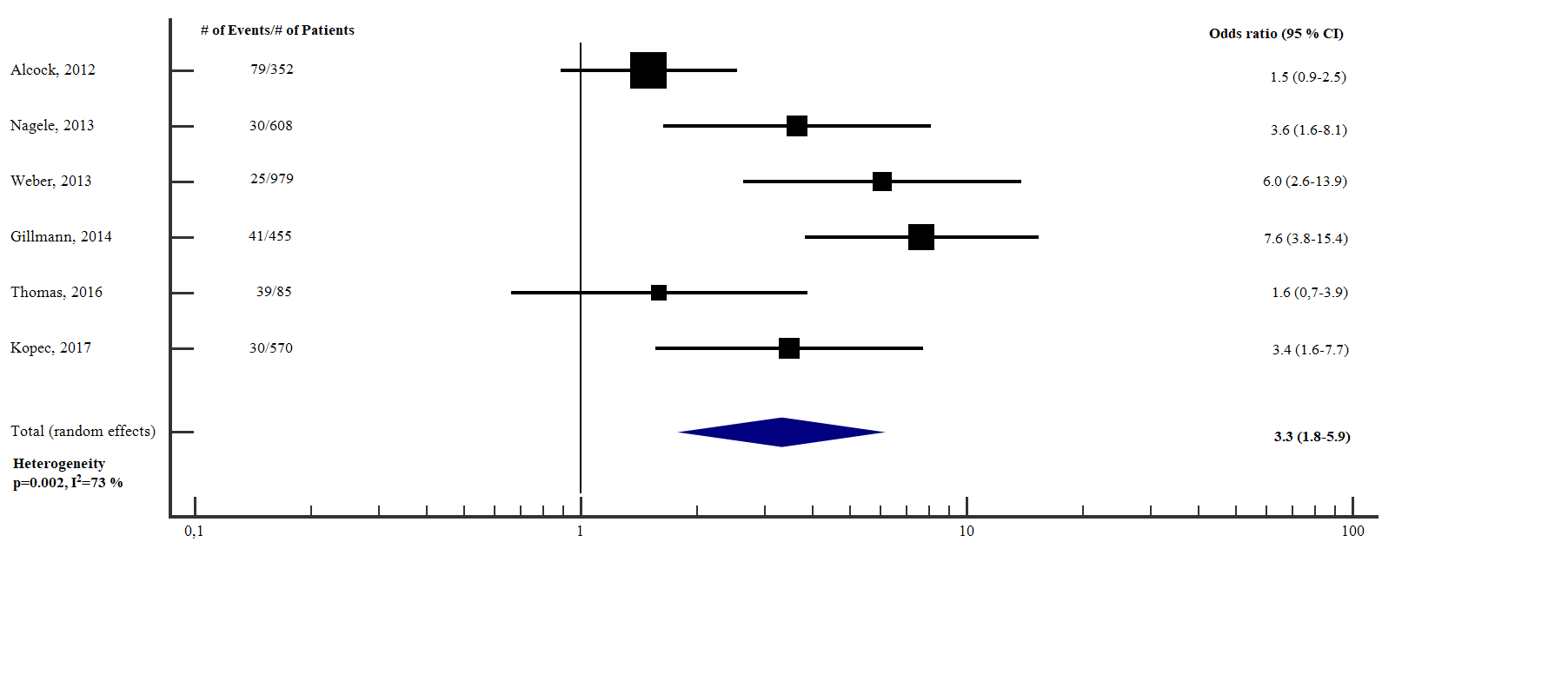

Supplement: S2 Fig — Forest plot showing the individual and pooled unadjusted odds ratios from the included studies. cTn = Cardiac troponin. CI = Confidence interval. # = Number of. (TIF) [file pone.0215094.s007.tif]

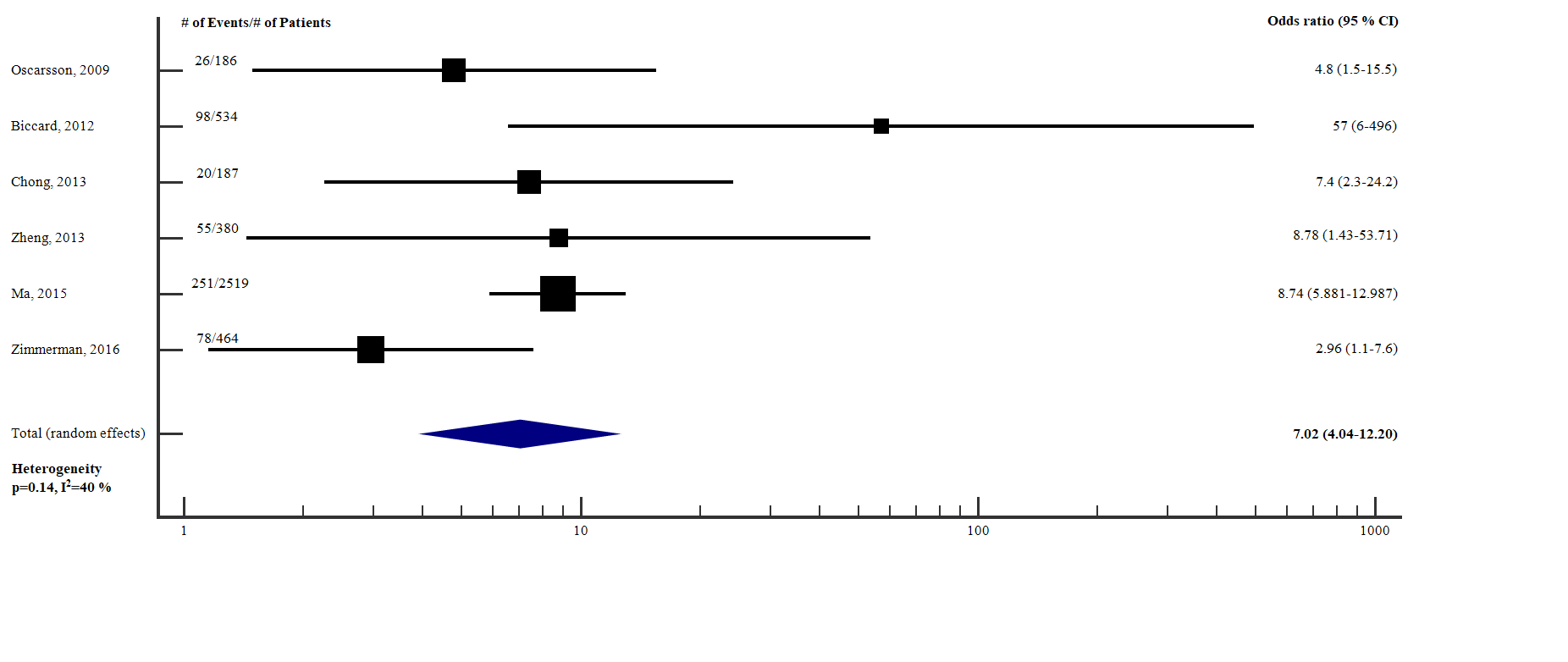

Supplement: S3 Fig — Forest plot showing the individual and pooled adjusted odds ratios from the included studies. cTn = Cardiac troponin. CI = Confidence interval. # = Number of. (TIF) [file pone.0215094.s008.tif]

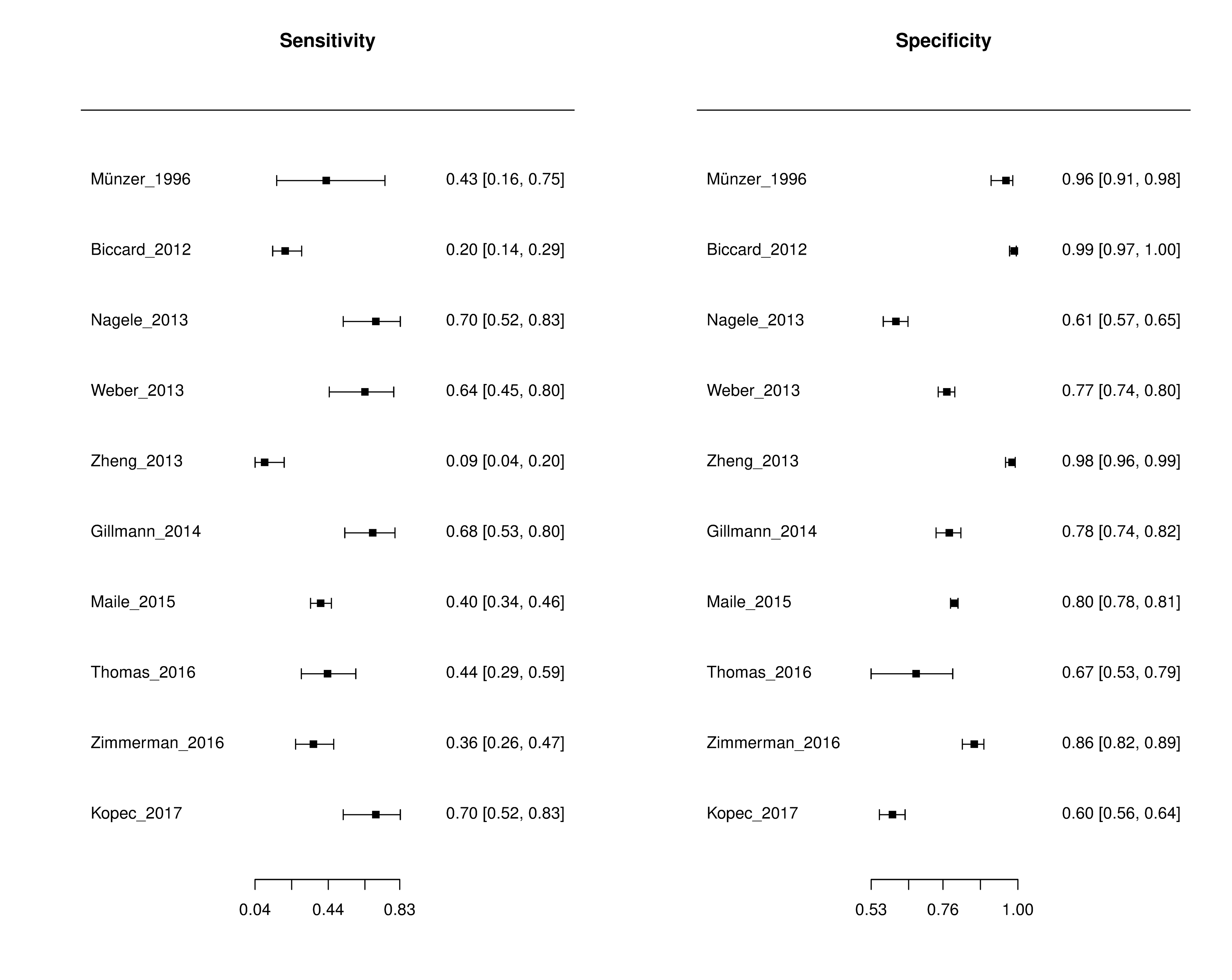

Supplement: S4 Fig — (TIF) [file pone.0215094.s009.tif]
